# Supplementary material for: Crystal Structure of the Cul2-Rbx1-EloBC-VHL Ubiquitin Ligase Complex
Source: Structure. 2017 Jun 6;25(6):901–911.e3. doi: 10.1016/j.str.2017.04.009 (PMC5462531; doi:10.1016/j.str.2017.04.009)
Supplement: Document S1. Figures S1–S4 and Tables S1–S6 [file mmc1.pdf]

Structure, Volume *25*

Supplemental Information

Crystal Structure of the Cul2-Rbx1-EloBC-VHL Ubiquitin Ligase Complex

Teresa A.F. Cardote, Morgan S. Gadd, and Alessio Ciulli

Supplemental Information

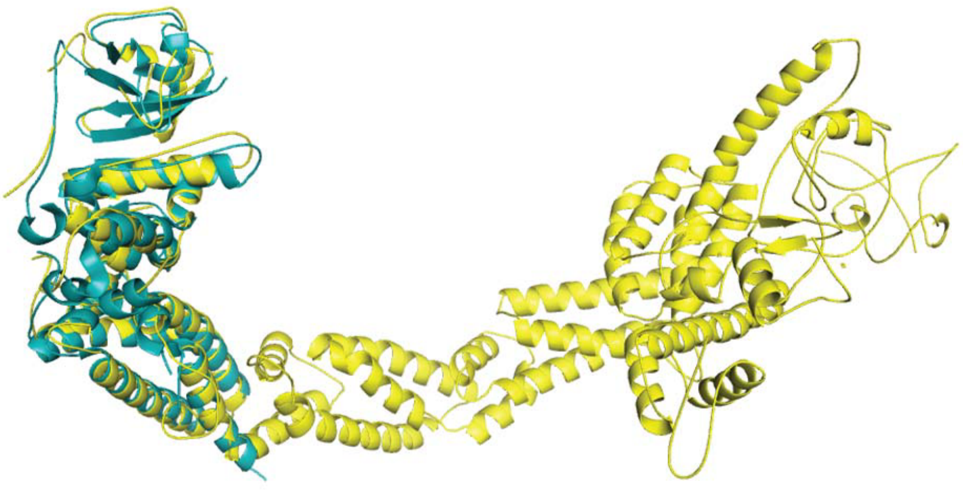

Figure S1. Related to Figure 1. Superposition of Cul2$_{1-163}$–VBC and CRL2^{VHL}. – The structure of the CRL2^{VHL} complex (PDB 5N4W) is superposed with the structure of VBC bound to Cul2$_{NTD}$ (PDB 4WQO). The structures align with a rmsd of 0.866 Å over the Cα atoms.

Table S1. Related to Figure 1. – Comparison of the areas of interface between Cul2 and VBC in the two structures available.

	**Buried surface area involving Cul2 interfaces (Å^2)**		
**PDB**	**pVHL-Cul2**	**EloC-Cul2**	**Total**
4WQO	322	944	1270
5N4W	328	1022	1350

Table S2. **Related to Figure 5.** – Buried surface area in the cullin interface with the receptor domains in three CRL complexes. PDBs 5N4W, 4JGH and 4N9F for $CRL2^{VHL}$, $CRL5^{SOCS2}$ and $CRL5^{Vif}$, respectively. The areas were calculated with GetArea (Fraczkiewicz & Braun 1998).

	Area (Å^2)		
	Polar	Apolar	Total
VBC	5556	7915	13471
Cul2	17339	26443	43782
Cul2-VBC	21997	32635	54632
ΔSASA	-899	-1723	-2621
SBC	7270	10909	18179
$Cul5_{NTD}$	7710	11709	19418
$Cul5_{NTD}$-SBC	14361	21291	35652
ΔSASA	-619	-1326	-1945
VifCβFBC	10068	14102	24170
$Cul5_{NTD}$	6540	9737	16278
$Cul5_{NTD}$-VifCβFBC	16085	22757	38842
ΔSASA	-523	-1082	-1605

Table S3. **Related to Figure 5. –** Comparison between experimental and theoretical Δ*Cp* values (Prabhu & Sharp 2005) for the Cul2–VBC interaction.

	Source	Δ*Cp* (cal/mol/K)
Experimental		-760
Theoretical	Spolar et al.	-425
	Murphy & Friere	-540
	Myers et al.	-400
	Makhatadze & Privalov	-692
	Robertson & Murphy	-384

Figure S2. Related to Figure 6. – AlphaLISA raw data of the titration of VBC and Cul2 mutants as displacers. The experiments were performed in quadruplicate and the results are an averaged value. The error bars represent the standard deviation of each point. The fitting was performed with GraphPad Prism 7 software.

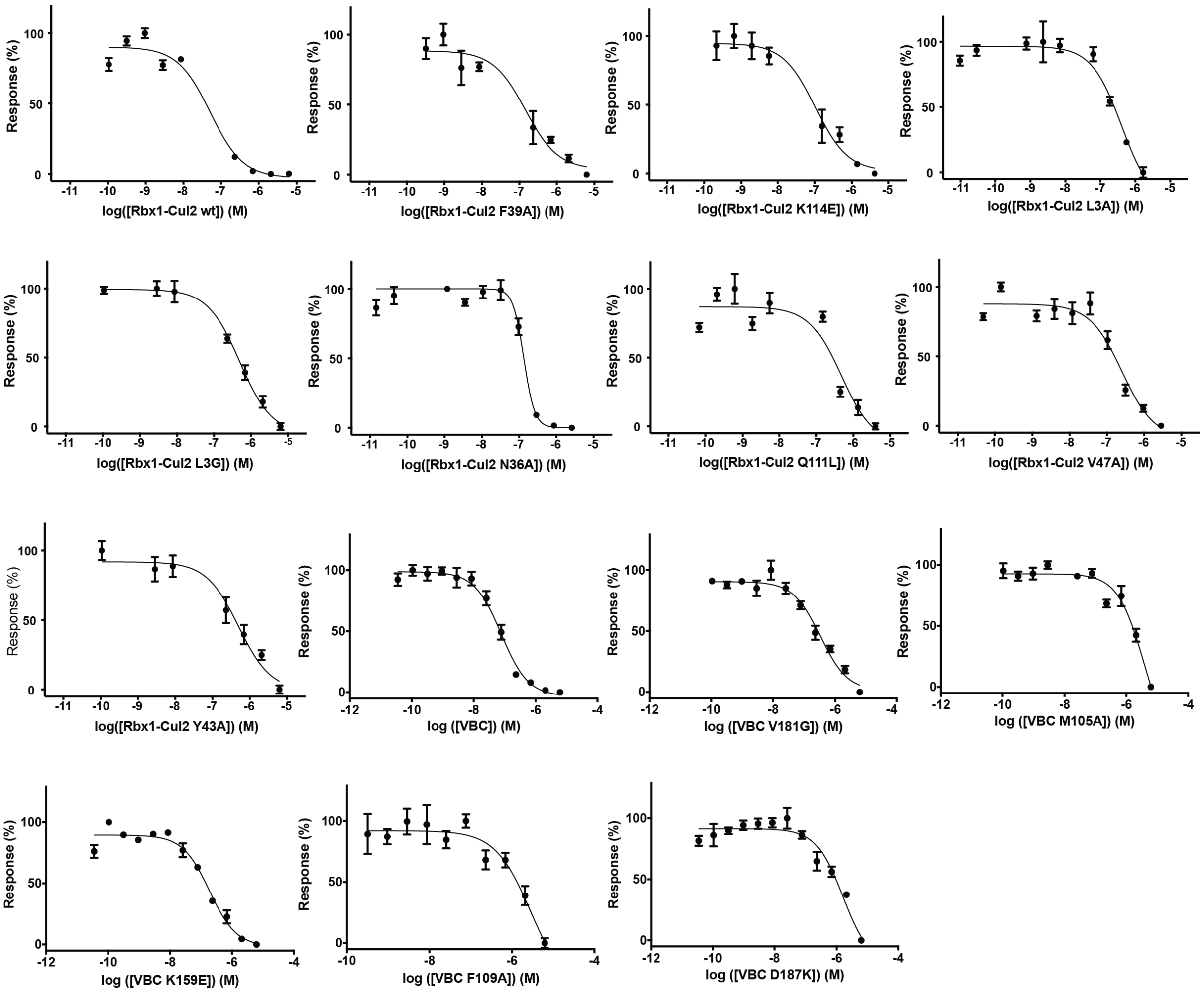

Table S4. Related to Figure 6 and Figure S2. – Alphalisa data of the titration of VBC and Cul2 mutants as displacers.

Displacer	IC_{50} (M)	95% CI	R^2	pIC_{50} (M)
VBC wt	7.80E-08	5.65E-08 to 1.08E-07	0.9550	-7.11 ± 0.07
VBC K159E	1.87E-07	1.34E-07 to 2.63E-07	0.9504	-6.73 ± 0.07
VBC D187K	1.59E-06	8.52E-07 to 2.97E-06	0.8773	-5.80 ± 0.13
VBC V181G	3.34E-07	2.21E-07 to 5.05E-07	0.9339	-6.48 ± 0.09
VBC M105A	3.90E-06	1.89E-06 to 8.02E-06	0.8978	-5.41 ± 0.15
VBC F109A	2.89E-06	1.40E-06 to 5.94E-06	0.8796	-5.54 ± 0.15
Cullin-2 wt	5.16E-08	2.77E-08 to 9.63E-08	0.9629	-7.29 ± 0.13
Cullin-2 L3A	4.08E-07	2.11E-07 to 7.91E-07	0.8816	-6.39 ± 0.14
Cullin-2 L3G	2.88E-07	1.26E-07 to 6.57E-07	0.8353	-6.54 ± 0.18
Cullin-2 P5A	8.11E-07	1.46E-07 to 4.50E-06	0.8302	-6.09 ± 0.36
Cullin-2 N36A	1.97E-07	1.16E-07 to 3.36E-07	0.9000	-6.71 ± 0.11
Cullin-2 F39A	1.59E-07	5.14E-08 to 4.91E-07	0.7952	-6.80 ± 0.24
Cullin-2 Y43A	2.40E-07	7.94E-08 to 7.27E-07	0.7529	-6.62 ± 0.24
Cullin-2 V47A	2.54E-07	1.43E-07 to 4.51E-07	0.8869	-6.59 ± 0.12
Cullin-2 Q111L	4.74E-07	2.27E-07 to 9.87E-07	0.8509	-6.32 ± 0.16
Cullin-2 K114E	1.04E-07	4.51E-08 to 2.41E-07	0.8798	-6.98 ± 0.18

Figure S3. Related to Table 3. – Isothermal Titration Calorimetry raw data of the titrations of VBC mutants versus Rbx1–Cul2.

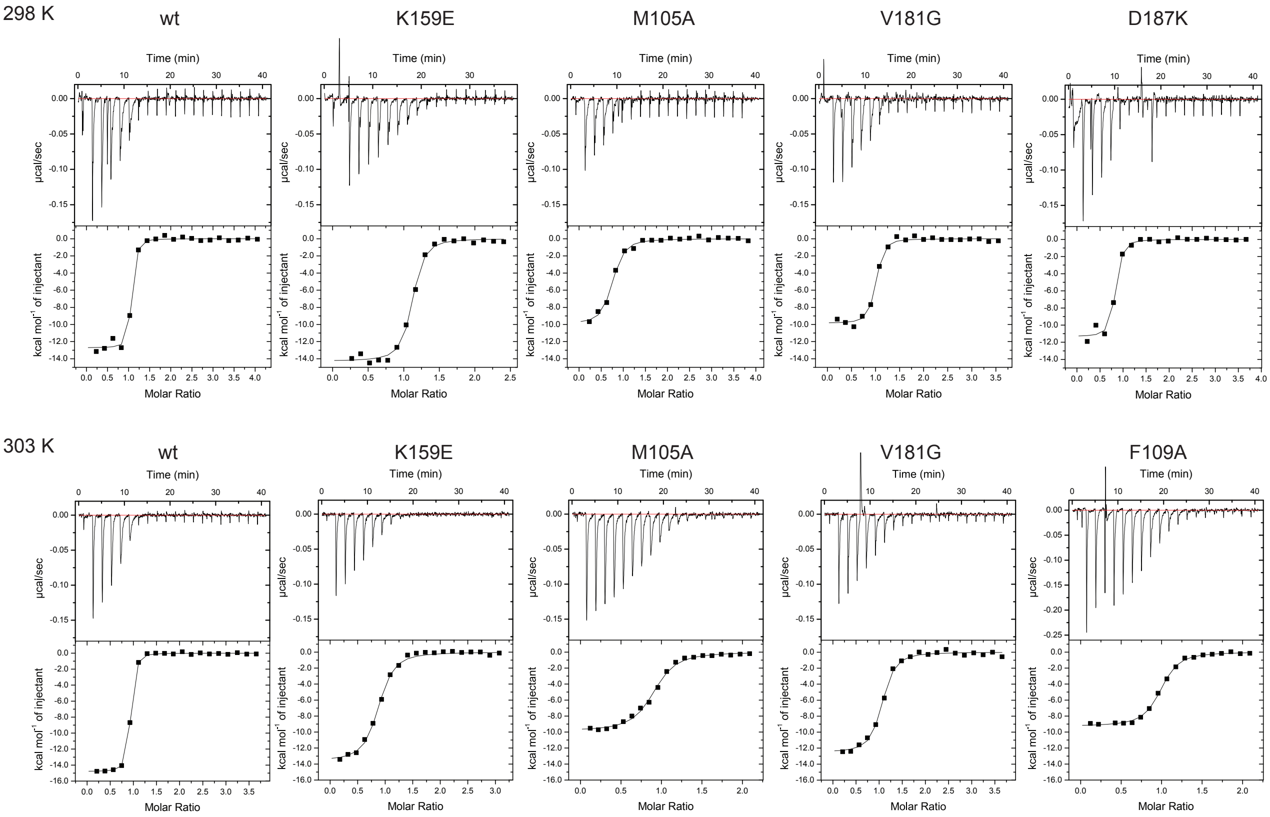

Figure S4. Related to Figure 7. Isothermal titration calorimetry data. – 200 μM of titrant diluted into 20 μM of titrate over 19 injections of 2 μl at 303 K. **a)** Titration of $Cul5_{NTD}$ into SBC and $S^{KSD}BC$. **b)** Titration of $V^{QRY}BC$ and VBC into Rbx1-Cul2.

a) **b)**

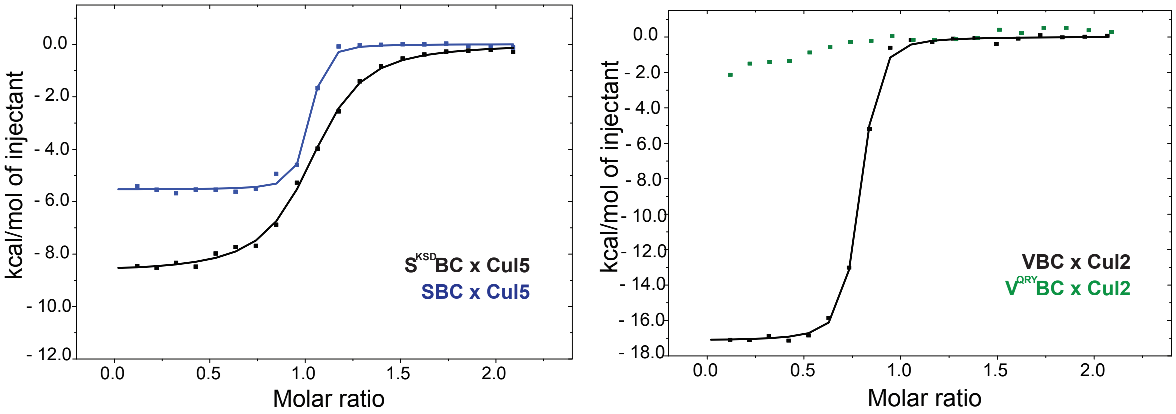

Table S5. Related to Figure 7 and Figure S4. – Isothermal Titration Calorimetry processed data of the experiments of the swapped domains. 150 μM of titrant diluted into 15 μM of titrate over 19 injections of 2 μl at 303 K. The errors refer to the fitting function.

	K_D (M)	ΔG (cal/mol)	ΔH (cal/mol)	TΔS (cal/mol)
VBC x Cul2	4.2×10^{-8} ± 5.0×10^{-9}	-10068 ± 70	-17210 ± 113	-7142 ± 133
V^{QRY}BC x Cul2	ND	ND	ND	ND
SBC x Cul2	ND	ND	ND	ND
S^{KSD}BC x Cul2	2.5×10^{-6} ± 2.7×10^{-7}	-7652 ± 64	-12980 ± 524	-5328 ± 528
VBC X Cul5	ND	ND	ND	ND
V^{QRY}BC x Cul5	2.9×10^{-6} ± 3.0×10^{-7}	-7554 ± 59	-7400 ± 386	154 ± 39
SBC x Cul5	9.8×10^{-9} ± 2.1×10^{-9}	-10936 ± 124	-5161 ± 33	5775 ± 128
S^{KSD}BC x Cul5	2.3×10^{-7} ± 3.2×10^{-8}	-9058 ± 81	-8354 ± 109	704 ± 136

Table S6. Related to STAR Methods. – Primers used for the mutations in Cullin-2, pVHL, Elongin C and SOCS2.

Protein	Mutation	Forward primer 5' -> 3'	Reverse primer 5'->3'
Cullin-2	L3A	CAGGGCGGATCCATGTCTGCGAAACCAAGAGTAGTAG	CTACTACTCTTGGTTTCGCAGACATGGATCCGCCCTG
	L3G	GTTCCGCGTGGATCCATGTCTGGGAAACCAAGAGTAGTAG	CTACTACTCTTGGTTTCCCAGACATGGATCCACGCGGAAC
	P5A	CCGCGTGGATCCATGTCTTTGAAAGCAAGAGTAGTAGATTTTGATGAAACATGG	CCATGTTTCATCAAAATCTACTACTCTTGCTTTCTCAGACATGGATCCACGCGG
	N36A	GGAATACGTCGAAAGAGCAACATGGGCTGACCGTTTCTCAGATATCTATGCTTTATG	CATAAAGCATAGATATCTGAGAAACGGTCAGCCCATGTTGCTCTTTCGACGTATTCC
	F39A	CGTCGAAAGAGCAACATGGAATGACCGTGCCTCAGATATCTATGCTTTATGTGTGGCC	GGCCACACATAAAGCATAGATATCTGAGGCACGGTCATTCCATGTTGCTCTTTCGACG
	Y43A	GGAATGACCGTTTCTCAGATATCGCTGCTTTATGTGTGGCCTATCCTGAACCCC	GGGGTTCAGGATAGGCCACACATAAAGCAGCGATATCTGAGAAACGGTCATTCC
	V47A	CAGATATCTATGCTTTATGTGCGGCCTATCCTGAACCCCTTGGAG	CTCCAAGGGGTTCAGGATAGGCCGCACATAAAGCATAGATATCTG
	Q111L	GGTATCTCAACACCCTGTTTATTAAAAAGAATAAATTAACAGAAGCGGACC	GGTCCGCTTCTGTTAATTTATTCTTTTTAATAAACAGGGTGTTGAGATACC
	K114E	GTATCTCAACACCCAGTTTATTGAAAAGAATAAATTAACAGAAG	CTTCTGTTAATTTATTCTTTTCAATAAACTGGGTGTTGAGATAC
pVHL	K159E	CACTGCCAGTGTATACTCTGGAAGAGCGATGCCTCCAGGTTGTCC	GGACAACCTGGAGGCATCGCTCTTCCAGAGTATACACTGGCAGTG
	K159Q	CCAATTGAGATCCCTAACAATCTAAGGCGTTCGCAGCCATCAGCAGTTCC	CCAATTGAGATCCCTAACAATCTAAGGCGTTCGCAGCCATCAGCAGTTCC
	V181G	GAATTACAGGAGACTGGACATCGGCAGGTCGCTCTACGAAGATC	GATCTTCGTAGAGCGACCTGCCGATGTCCAGTCTCCTGTAATTC
	S183R+D187Y	CCAATTGAGATCCCTAACAATCTAAGGCGTTCGCAGCCATCAGCAGTTCC	CGTCAGGCGGCTCTACGAATATCTGGAAGACCACCCAAATGTGC
	D187K	CATCGTCAGGTCGCTCTACGAAAAACTGGAAGACCACCCAAATG	CATTTGGGTGGTCTTCCAGTTTTTCGTAGAGCGACCTGACGATG
EloC	M105A	GAAATTGCACTGGAACTGCTGGCGGCTGCGAACTTCTTAGATTGTTAG	CTAACAATCTAAGAAGTTCGCAGCCGCCAGCAGTTCCAGTGCAATTTC
	F109A	GGAACTGCTGATGGCTGCGAACGCCTTAGATTGTTAGGGATCTCAATTGG	CCAATTGAGATCCCTAACAATCTAAGGCGTTCGCAGCCATCAGCAGTTCC
SOCS2	Q164K	CCGCTCTACACGTCAGCACCATCTCTGAAGCATCTCTGTAGGCTCACCATTAAC	GTTAAGTGTGAGCCTACAGAGATGCTTCAGAGATGGTGCTGACGTGTAGAGCGG
	R186S+Y190D	CATCTGGGGACTGCCTTTACCAACAAGCCTAAAAGATGACTTGGAAG	CTTCCAAGTAATCTTTTAGGCTTGTTGGTAAAGGCAGTCCCCAGATG
