# Supplementary material for: Crystal Structure of the Cul2-Rbx1-EloBC-VHL Ubiquitin Ligase Complex
Source: Structure. 2017 Jun 6;25(6):901–911.e3. doi: 10.1016/j.str.2017.04.009 (PMC5462531; doi:10.1016/j.str.2017.04.009)
Supplement: Document S2. Article plus Supplemental Information [file mmc2.pdf]

Structure

Article

Crystal Structure of the Cul2-Rbx1-EloBC-VHL Ubiquitin Ligase Complex

Graphical Abstract

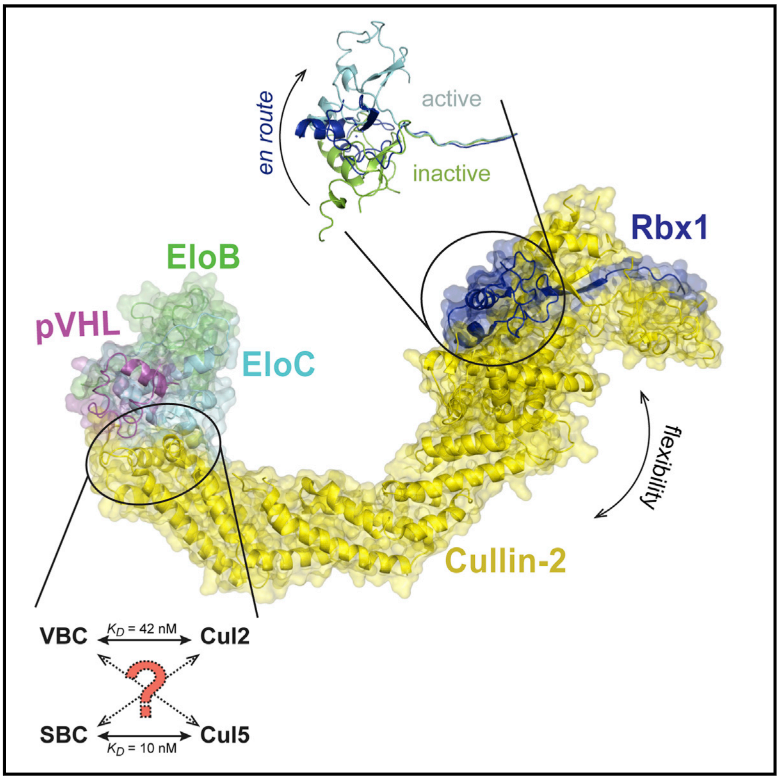

Authors

Teresa A.F. Cardote, Morgan S. Gadd, Alessio Ciulli

Correspondence

a.ciulli@dundee.ac.uk

In Brief

Cardote et al. describe a crystal structure of a pentameric CRL2^{VHL} complex consisting of Cul2, Rbx1, Elongin B, Elongin C, and pVHL. The structure traps a closed state of full-length Cul2, and additional biophysical characterization of the binding properties identifies features that contribute to Cul2 versus Cul5 recognition.

Highlights

- First crystal structure of the CRL2^{VHL} complex
- Full-length structure of Cullin-2 interacting with Rbx1
- Suggested trajectory of the Rbx1 RING domain en route to the fully active E3 ligase
- Biophysical data reveal basis for Cul2 versus Cul5 recognition

Data Resources

5N4W

Cardote et al., 2017, Structure *25*, 901–911
June 6, 2017 © 2017 The Author(s). Published by Elsevier Ltd.
http://dx.doi.org/10.1016/j.str.2017.04.009

CellPress

Crystal Structure of the Cul2-Rbx1-EloBC-VHL Ubiquitin Ligase Complex

Teresa A.F. Cardote,[1] Morgan S. Gadd,[1] and Alessio Ciulli[1,2,*]
[1]Division of Biological Chemistry and Drug Discovery, School of Life Sciences, University of Dundee, Dow Street, Dundee DD1 5EH, UK
[2]Lead Contact
*Correspondence: a.ciulli@dundee.ac.uk
http://dx.doi.org/10.1016/j.str.2017.04.009

SUMMARY

Cullin RING E3 ubiquitin ligases (CRLs) function in the ubiquitin proteasome system to catalyze the transfer of ubiquitin from E2 conjugating enzymes to specific substrate proteins. CRLs are large dynamic complexes and attractive drug targets for the development of small-molecule inhibitors and chemical inducers of protein degradation. The atomic details of whole CRL assembly and interactions that dictate subunit specificity remain elusive. Here we present the crystal structure of a pentameric CRL2^{VHL} complex, composed of Cul2, Rbx1, Elongin B, Elongin C, and pVHL. The structure traps a closed state of full-length Cul2 and a new pose of Rbx1 in a trajectory from closed to open conformation. We characterize hotspots and binding thermodynamics at the interface between Cul2 and pVHL-EloBC and identify mutations that contribute toward a selectivity switch for Cul2 versus Cul5 recognition. Our findings provide structural and biophysical insights into the whole Cul2 complex that could aid future drug targeting.

INTRODUCTION

In living systems, complex signaling mechanisms and networks continuously regulate cells. The ubiquitin proteasome system contributes significantly to these regulation processes by determining the fate of many proteins under different cellular circumstances. Protein tagging with ubiquitin molecules translates into a variety of cellular responses that are dictated by the pattern of ubiquitination, including ubiquitin-dependent proteasomal degradation (Hershko and Ciechanover, 1998). The ubiquitination pathway relies on the sequential action of three enzymes: an E1-activating enzyme, an E2-conjugating enzyme, and an E3 ligase. Cullin RING E3 ubiquitin ligases (CRLs) constitute the major subfamily of E3 ligases that catalyze the transfer of ubiquitin from the E2-conjugating enzyme to the target substrate. CRLs account for 20% of the ubiquitin-dependent protein turnover in cells (Petroski and Deshaies, 2005). The Cullin protein is the core of CRLs, acting as a scaffold that brings together the substrate and the E2-conjugating enzyme. The substrate is recruited by the receptor and the E2 enzyme loaded with ubiquitin is recruited by the RING finger protein (Lydeard et al., 2013). The Cullin family is composed of seven members involved in ubiquitin multi-subunit complexes (Cul1, Cul2, Cul3, Cul4A, Cul4B, Cul5, and Cul7) which all share similar structural features (Duda et al., 2011). To date, only Cul1 (Zheng et al., 2002), Cul4A, and Cul4B (Fischer et al., 2011) have yielded crystal structures of full-length protein, limiting the information available on this important protein family. Therefore, the only structures available of whole CRL complexes are those of CRL4A^{DDB2}, CRL4B^{DDB2}, and CRL1^{Skp2}. Limited structural information prevents full comprehension of mechanisms of activity and functioning of CRL multi-subunit molecular machines. Structural information on CRLs is critical also for drug discovery as it can provide the basis for exploring protein-protein interactions (PPIs) with small molecules to modulate protein function (Lucas and Ciulli, 2017). For example, based on the structural knowledge of certain PPIs, recent work has demonstrated the ability to hijack E3 ligase complexes into recruiting non-natural selective substrates for ubiquitination and subsequent proteasomal degradation (Lu et al., 2015; Winter et al., 2015; Zengerle et al., 2015). These examples reinforce the importance of available structural information but also demonstrate the power of targeting CRL complexes using small molecules.

Our work focuses on the CRL2^{VHL} ligase, a complex containing Cul2 as the scaffold protein and the von Hippel-Lindau protein (pVHL) as the substrate receptor. Cul2 recruits pVHL at its N-terminal region through an adaptor subunit constituted by a dimeric complex formed by Elongin B (EloB) and Elongin C (EloC) (Pause et al., 1997), and the RING finger subunit Rbx1 at its C-terminal region (Kamura et al., 1999). Despite the existence of crystal structures of partially assembled complexes, namely, structures of the trimeric complex constituted by pVHL and EloBC (VBC) (Stebbins et al., 1999), VBC in complex with a hypoxia-inducible factor 1α (HIF-1α) peptide (Min et al., 2002; Hon et al., 2002), and the first helical bundle of Cul2 (residues 1–163) bound to VBC (Nguyen et al., 2015), the structure of the whole CRL2^{VHL} complex was still elusive. CRL2^{VHL}-mediated degradation of HIF-1α is the most widely studied function of this E3 ligase; however, there are at least another six substrates of the VHL ubiquitin ligase that have been identified (Cai and Yang, 2016). Under normal levels of oxygen (normoxia), HIF-1α is hydroxylated by oxygen-dependent prolyl hydroxylase domain (PHD) enzymes at specific proline residues (Pro402 and Pro564). As a result, HIF-1α is recruited to the CRL2^{VHL} complex via the β domain of pVHL, which recognizes the hydroxyproline post-translation modification. Once bound to CRL2^{VHL}, HIF-1α is ubiquitinated and targeted for degradation by the

This is an open access article under the CC BY license (http://creativecommons.org/licenses/by/4.0/).

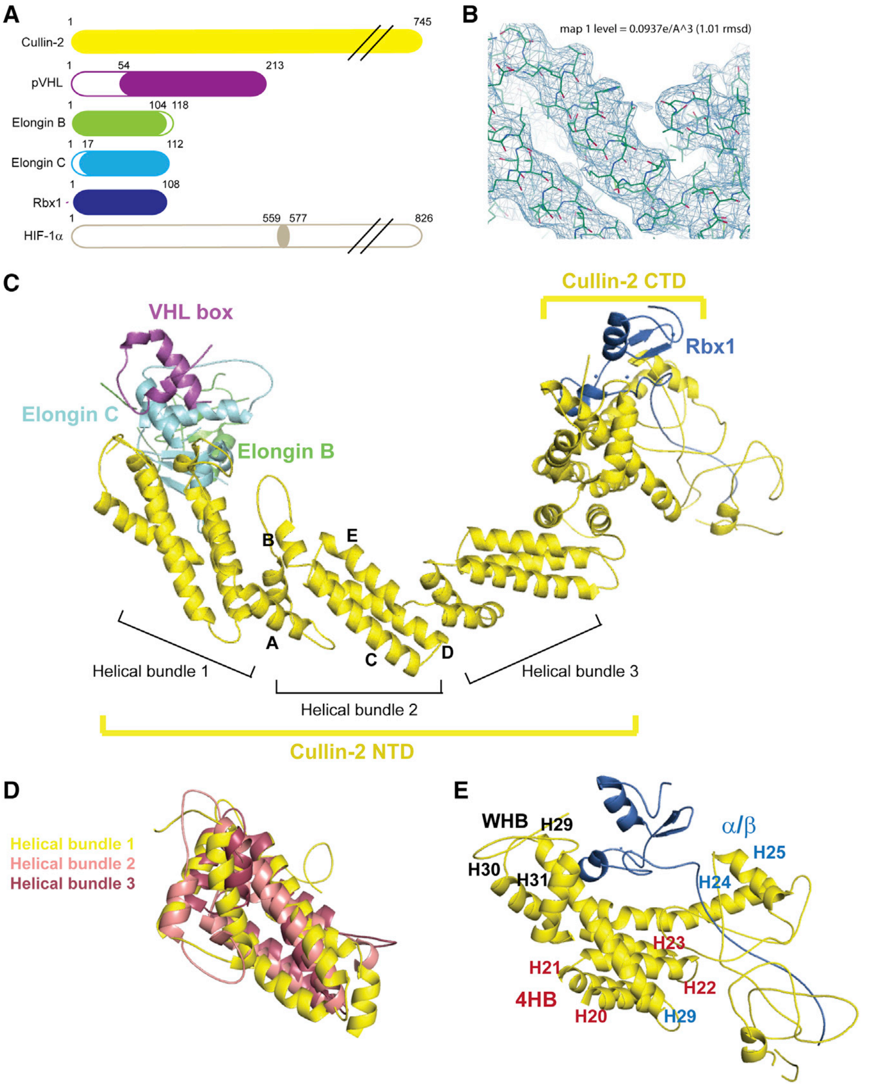

Figure 1. Crystal Structure of the CRL2^{VHL} Complex

(A) Diagrams of the primary structure of each protein subunit, illustrating the constructs used to obtain the crystal structure.

(B) Representation of the $2F_o$-F_c electron density contoured at 1σ over a portion of the model.

(C) Crystal structure of the CRL2^{VHL} with each of the protein subunits identified. Cul2 is divided into N-terminal (NTD) and C-terminal (CTD) domains and the NTD is composed of three helical bundles, constituted by five α-helices (A–E).

(D) The three helical bundles of the NTD are superposable with a maximum RMSD of 4.5 Å over the Cα.

(E) CTD of Cul2 is organized in a four-helical bundle (4HB), an α/β domain, and a winged-helix motif (WH-B).

proteasome (Maxwell et al., 1999). Upon decrease of the oxygen levels, PHD activity is inhibited and HIF-1α is no longer hydroxylated, escapes E3 recognition, and accumulates in the cell, triggering a transcriptional response to hypoxia (Semenza, 2007). The CRL2^{VHL} is considered an attractive therapeutic target in diseases such as chronic anemia and acute ischemic disorders where the effects of stabilization of HIF-1α in cells have proved beneficial (Muchnik and Kaplan, 2011). Our laboratory has recently developed potent pVHL inhibitors using structure-guided drug design (Galdeano et al., 2014). Compound VH298 was characterized in cells as highly selective inhibitor active on-target against pVHL, and elected as a chemical probe of the hypoxia signaling pathway (Frost et al., 2016). Using these pVHL-targeting ligands as a starting point, bivalent molecules have been designed which recruit proteins into proximity of the CRL2^{VHL} to deplete protein levels inside cells and in vivo (Zengerle et al., 2015). Targeting CRL2^{VHL} to induce protein degradation provides an attractive chemical biology approach for target validation (Buckley et al., 2015; Fulcher et al., 2016) and a new therapeutic modality in drug discovery (Lai and Crews, 2017).

Here, we present the first crystal structure of the CRL2^{VHL} complex to 3.9 Å resolution, revealing for the first time the full-length Cul2 interacting with Rbx1, which provides insights to the mechanism of the E3 ligase activity. We complement our study with biophysical data on CRL2^{VHL} PPIs, revealing the basis for Cul2 versus Cul5 recognition.

RESULTS

Crystal Structure of the CRL2^{VHL} Complex

To gain structural insight into the human CRL2^{VHL} complex, we reconstituted the fully assembled complex composed by Cul2, EloB, EloC, pVHL, and Rbx1 and pursued its crystal structure (Figure 1). To assemble the complex, we expressed and purified independently a trimeric complex constituted by pVHL, EloB and EloC (i.e., VBC) and a dimeric complex composed by Cul2 and Rbx1. The first complex is obtained routinely in the *Escherichia coli* expression system (Van Molle et al., 2012), whereas the latter was expressed in *Sf21* insect cells (Bulatov et al., 2015; Kelsall et al., 2013). The two components VBC and Rbx1-Cul2 were mixed and purified by size-exclusion chromatography, yielding the pentameric complex. In parallel, we prepared the same complex in the presence of an HIF-1α 19-mer peptide (residues 559–577) bound to pVHL, mimicking one of the natural substrates of the CRL2^{VHL} ubiquitin ligase. Sparse-matrix screening of both complex forms was performed and hit conditions were identified for the HIF-1α containing form. After optimization of the initial hit condition by additive screening and the introduction of streak seeding, we obtained promising diffracting crystals to be used for data collection. These crystals exhibited some elasticity and were to some extent fragile; nevertheless, we collected a dataset for crystals of the CRL2^{VHL} complex. Calculation of the Matthews coefficient (Matthews, 1968) retrieved a solvent percentage of 63.9%,

Table 1. Data Collection and Refinement Statistics

Data Collection	
Wavelength (Å)	0.9282
Space group	$C222_1$
Cell dimensions	
a, b, c (Å)	86.0, 191.0, 238.9
α, β, γ (°)	90, 90, 90
Molecules/ASU	1
Resolution (Å)	95.48–3.90 (4.27–3.90)
R_{merge} (%)	11.4 (73.2)
$I/\sigma(I)$	10.2 (2.2)
Completeness (%)	100 (100)
Redundancy	5.7 (5.3)
$CC_{1/2}$	0.857 (0.798)
Refinement	
Resolution (Å)	95.48–3.9
Unique reflections	18,326
R_{work}/R_{free} (%)	30.15/34.61
Average B factor (Å^2)	191
No. of non-hydrogen atoms	7,734
RMSDs	
Bond lengths (Å)	0.003
Bond angles (°)	0.692
Ramachandran analysis	
Preferred regions (%)	90.65
Allowed regions (%)	9.13
Outliers (%)	0.22

Statistics in parentheses indicate values for the highest-resolution shell.

consistent with the presence of one protomer per asymmetric unit (ASU) arranged in a $C222_1$ space group (Table 1). The structure was solved at 3.9 Å resolution using a combination of molecular replacement (MR) and iterative rounds of model building and refinement, which are described next. The structure of Cul2 was the first fragment to be solved by MR in MrBUMP (Keegan and Winn, 2007), using an homology model of the same protein obtained through Chimera (Pettersen et al., 2004) based on the structures of Cul1, Cul4, $Cul5_{NTD}$, and $Cul5_{CTD}$ as template (PDB: 1U6G, 4A0K, 4JGH, and 3DPL, respectively). Next, we placed the dimer of EloB and EloC (EloBC) by MR in Phaser (McCoy et al., 2007), using as template the corresponding subunits from a VBC-HIF-1α structure (PDB: 4AJY). Clear unmodeled electron density could be observed corresponding to the Rbx1 N-terminal tail (residues 17–35). This region is conserved in all Cullin-bound Rbx1 structures available in the PDB, allowing its correct positioning into the structure. We next identified the RING domain of Rbx1 by MR using as template an existing Rbx1 structure (PDB: 2LGV [Spratt et al., 2012]) in Phaser. At this stage, patches of positive electron density became clearly visible in the area where pVHL was expected to be found, according to the previous structure of VBC in complex with $Cul2_{1-163}$ (Nguyen et al., 2015). Fitting in pVHL proved very challenging and despite various attempts at MR using a diversity of template models, no satisfactory solution was found. However, due to the unambiguous electron density, we were able to fit manually the three-helix cluster structure of the VHL box (Kamura et al., 2004) that is conserved in all the available crystal structures. Nevertheless, for the remaining residues (54–156) that constitute the substrate-binding domain and the 19-mer HIF-1α peptide, no electron density was observed. Due to the resolution of our structure, not all the side chains could be definitively seen in the electron density; therefore some side chains, particularly in the Cul2-VBC interface, were modeled according to a higher-resolution structure (Nguyen et al., 2015). These steps were intercalated with rounds of refinement in Refmac5 (Vagin et al., 2004) and Phenix (Adams et al., 2010), and map sharpening by negative B-factor correction was applied to improve the quality of the electron density maps and facilitate successful model building (Figure 1B) (Nicholls et al., 2012). The structure packs nicely with eight symmetry-related molecules, forming extensive crystal contacts. The full-length Cul2 binding Rbx1 at the C-terminal domain and the VHL-box-EloBC at the N-terminal domain constitute our final model (Figure 1C). It reveals for the first time the whole Cul2 structure and the interface between Rbx1 and Cul2.

Cullin-2 Structure Highlights Its Inherent Flexibility

The structure assumed by Cul2 in the $CRL2^{VHL}$ reveals an architecture similar to that of structures of other Cullins. It presents the classical elongated shape divided in the N-terminal domain (NTD) and the C-terminal domain (CTD). The NTD comprises residues 1–384 arranged in three helical bundles (also called Cullin repeats) each composed of five α helices. These three Cullin repeats are superposable with a root-mean-square deviation (RMSD) for the Cα atoms of up to 4.5 Å (Figure 1D). At the other end, the CTD (residues 385–745) is a globular domain organized in a four-helical bundle (4HB) linked to the NTD, an α/β domain, and a winged-helix motif (WH-B) (Figure 1E). In our structure, some residues at the CTD (625–634 and 645–660) were disordered and could not be modeled.

Cul2 was one of the members of the Cullin family lacking structural information on the full NTD. Superposition of all the existing structures of complete Cullin NTDs highlights the flexibility of this domain. We observed that all of the NTDs align most closely through the second Cullin repeat, exhibiting considerable differences in their first and third helical bundles' relative position, consistent with intra-domain bending motion (Figure 2A). In addition, the structural alignment of full-length Cul1, Cul2, and Cul4A reveals an interesting difference on how the CTD is packed against the NTD in each of the structures (Figures 2B and 2C). In Cul2 the globular domain is considerably moved toward the NTD relative to the other Cullins. The distances measured between the very top residue at the CTD (the Lys that gets modified with NEDD8, i.e., Lys689 in Cul2, Lys720 in Cul1, and Lys705 in Cul4) and the top residue in helix α1 of the NTD (Phe10 in Cul2, Leu17 in Cul1, and Thr62 in Cul4) are more than 10 Å shorter in Cul2 (100 Å for Cul2, 110 Å for Cul1, and 113 Å for Cul4). $Cul1_{CTD}$ is rotated by 29° compared with $Cul2_{CTD}$, whereas $Cul4_{CTD}$ presents an angle of rotation of about 33° compared with $Cul2_{CTD}$.

In summary, these observations highlight a trapped closed state conformation for non-neddylated full-length Cul2 and support the previously reported flexibility of the Cullin scaffold,

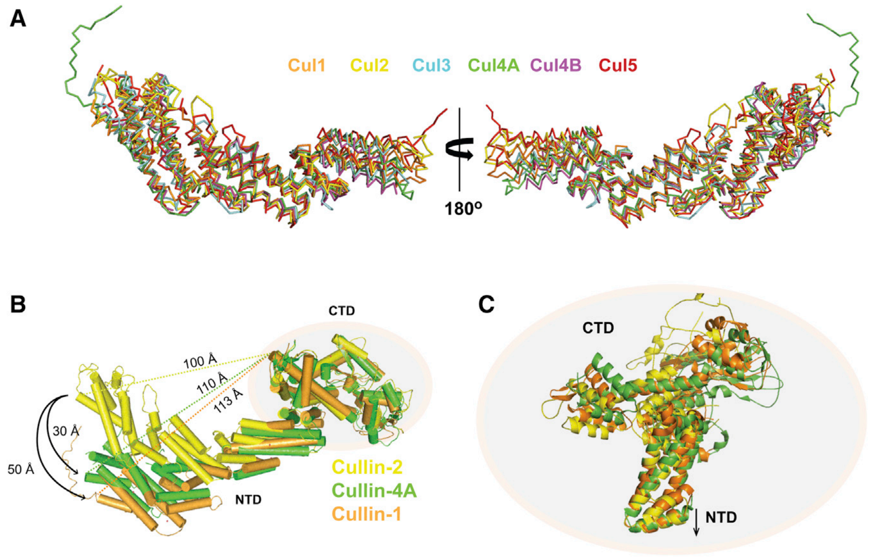

Figure 2. The CRL2^{VHL} Complex Is Dynamic and Flexible

(A) Crystal structures of the full NTD of Cul1 (PDB: 1U6G), Cul2 (PDB: 5N4W), Cul3 (PDB: 4HXI), Cul4A (PDB: 4A0K), Cul4B (PDB: 4A64), and Cul5 (PDB: 4JGH), illustrating the existence of hinge points in the linkers between Cullin repeats.

(B) Full-length structures of Cul2, Cul4A, and Cul1 superposed by the CTDs reveal considerable inter-domain flexibility through a hinge point between NTD and CTD.

(C) Close-up view of the CTDs of Cul1, Cul2, and Cul4A with the proteins aligned by the third Cullin repeat of the NTD, illustrating the different relative orientations of the CTDs in the full-length structures.

which is thought to contribute to an allosteric mechanism of polyubiquitination of substrates (Liu and Nussinov, 2011).

Rbx1 Presents a New Orientation

Rbx1 structure presents an N-terminal tail arranged in a long β strand that engages Cullin CTD and a variant RING finger domain. The RING is characterized by the presence of an extended region containing a third zinc ion coordinated by three cysteines and one histidine, in addition to the canonical region containing two zinc ions (Zheng et al., 2002). The structure of the N-terminal tail of Rbx1 and its position in the complex are conserved among different CRL structures. Conversely, the RING domain is flexible and has been found in a number of different orientations, even in different molecules within the same ASU (Duda et al., 2008). Interestingly, the conformation observed in our structure has not been reported previously. The Rbx1 RING domain establishes mainly hydrophobic crystal contacts with Rbx1-Cul2 from another ASU. These contacts extend through an area of 363 Å^2, which corresponds to about 17% of the total area of interface of Rbx1 with Cul2 (2,122 Å^2). Thus we believe that our observed conformation of Rbx1 captures, via the consequent crystal contacts, a significantly populated conformation in solution. It lies between Rbx1-Cul5∼NEDD8 and Rbx1-Cul5 conformations, resembling a transitory state in a trajectory amid the two (Figure 3). The flexibility of the RING domain has been the subject of many studies aimed at elucidating the structural and mechanistic details of ubiquitin ligase activity (Angers et al., 2006; Calabrese et al., 2011; Duda et al., 2008; Goldenberg et al., 2004; Onel et al., 2017; Zheng et al., 2002). Previous work has demonstrated how the RING domain dramatically reorients its position relative to Cullin upon neddylation of Cul5 (Scott et al., 2014). This repositioning is regarded as the enzyme adaptation for ubiquitination and, in this so-called open conformation, the area of interface between Rbx1 and Cul5 (1,707 Å^2) is contributed solely by the N-terminal tail (Table 2). Indeed the total areas of interface between Rbx1 and the different Cullin subunits vary according to the orientation of the Rbx1 RING domain. In our structure, the linker between the N-terminal tail and the RING domain, albeit not completely extended, is not fully retracted either, and the Rbx1-Cul2 PPI covers an area of 2,122 Å^2. This interface area is larger than in the Rbx1-Cul5∼N8 and the Glomulin-Rbx1-Cul1 (Duda et al., 2012) complexes but smaller than in any of the other structures (Table 2). These findings reveal a potential trajectory of the Rbx1 RING domain from the non-neddylated to neddylated form of the complex, en route to the fully active E3 ligase.

The Interface between Cul2 and VBC

The Cul2-VBC interface, as previously described (Nguyen et al., 2015), is established by three main contact points (Figure 4). Firstly, the N-terminal loop of Cul2 assumes a critical role in the interaction (Figure 4A), with Leu3 inserting into an hydrophobic pocket on EloC, defined by Met105 and Phe109, and Pro5 being involved in a three-way contact with Val181 in pVHL and Met105 in EloC (Figure 4B). The importance of the N-terminal loop in this PPI is observed in Cul2 and Cul3 (Canning et al., 2013). In a second region of interaction there is charge complementarity at the interface between helix α5 of Cullin and the substrate receptor, where residues Gln111 and Lys114 from Cul2 are interacting with residues Lys159 and Asp187 from pVHL, respectively. These two salt bridges were thought to be responsible for the selective recruitment of Cul2 to this complex over Cul5 (Nguyen et al., 2015). Finally, helix α2 of Cul2 is packed against pVHL and EloC surfaces, with residues Asn36, Phe39, Tyr43, and Val47 contributing to this hydrophobic interface (Figure 4D). EloB forms no direct interaction with Cul2. The areas of the Cul2-VBC PPI and the orientation of the VHL-box-EloBC relative to Cul2 are consistent with those observed in the quaternary structure of Cul2$_{1-163}$-VBC (Table S1 and Figure S1). The two structures superpose with an RMSD for the Cα atoms of 0.866 Å, suggesting this interface of the CRL complex to be structurally rigid and conserved.

Thermodynamics of Cul2-VBC Interaction

The lack of biophysical data regarding PPIs within the CRL2^{VHL} complex encouraged us to pursue in-depth characterization. Despite numerous attempts to obtain Cul2 alone as functional protein by testing different length constructs with different solubilizing tags, we failed to obtain soluble, monomeric protein that

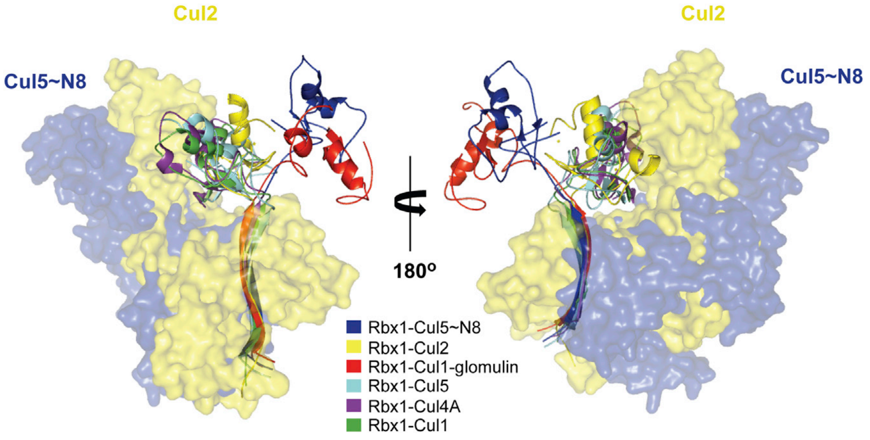

Figure 3. Rbx1 Presents a New Orientation

Superposition of the CTDs of six Cullins—Cul1 (PDB: 1U6G), Cul2 (PDB: 5N4W), Cul4 (PDB: 4A0C), Cul5 (PDB: 3DPL), Cul5∼NEDD8 (PDB: 3DQV), and Glomulin-Rbx1-Cul1 (PDB: 4F52)—complexed with Rbx1. The new structure of Rbx1 in complex with Cul2 unveils a novel orientation of its RING domain, resembling an en route conformation between Rbx1-Cul5 and Rbx1-Cul5∼NEDD8.

could form a complex with VBC. Since Cul2 needed to be co-expressed with Rbx1 to obtain soluble and functional protein, it was not possible to investigate the PPI between these two subunits, so we decided to focus on the PPI between Cul2 and VBC. We performed isothermal titration calorimetry (ITC) experiments titrating VBC into Rbx1-Cul2 and measured an expectedly strong binding affinity (K_D = 42 nM) and large exothermic binding enthalpy (ΔH = -17.2 kcal/mol) at 303 K (Figure 5). Previously published data on other Cul5-EloBC complexes, namely $Cul5_{NTD}$-ASB9-EloBC and $Cul5_{NTD}$-SOCS2-EloBC, reported binding affinities of 140–220 nM (Muniz et al., 2013; Thomas et al., 2013) and 7–47 nM (Babon et al., 2009; Bulatov et al., 2015; Salter et al., 2012) and binding enthalpies of -8.3 kcal/mol and -4.8 kcal/mol, respectively, for the corresponding interactions. We next investigated temperature dependence of the binding parameters and determined a change in heat capacity, ΔC_p = -760 cal/mol/K (Figure 5B). The change in heat capacity is a thermodynamic parameter defined as the change in energy (heat) with temperature ($\Delta C_p = \delta \Delta H/\delta T$) (Prabhu and Sharp, 2005). This value of ΔC_p is significantly greater than those determined for similar complexes (Figure 5C), suggesting a greater buried surface area upon interaction, leading to a more stable complex. In fact, based on the crystal structures, the area buried upon interaction in Cul2-VBC is 2,621 $Å^2$, which is considerably greater than in Cul5-SBC (1,945 $Å^2$) and Cul5-VifCβFBC (1,605 $Å^2$, Table S2). It has been shown that hydration of polar and apolar groups promotes changes in the heat capacity and this relation has been modeled by the equation $\Delta C_p^{hydration} = C_a \Delta SASA_a + C_p \Delta SASA_p$, where SASA is the solvent-accessible surface area (apolar and polar) buried upon interaction and C_a and C_p are constants empirically determined, representing the area coefficient per $Å^2$ contribution of residues in heat capacity change. Based on our crystal structure we calculated the theoretical ΔC_p values according to the different constants C_a and C_p (Table S3) (Prabhu and Sharp, 2005). Interestingly, we observed that our experimental ΔC_p is greater than the theoretical values in most of the cases, which could indicate a conformational rearrangement in solution upon binding that is not accounted for in the theoretical calculations.

The biophysical and structural data support a tight and extensive PPI between the scaffold and the adaptor/receptor subunits in the $CRL2^{VHL}$ complex.

Hydrophobic Contact Residues Critical for the Cul2-VBC Interaction

To elucidate the main drivers of the tight PPI between Cul2 and VBC and identify hotspots, we turned to quantifying individual contributions of potential key residues, guided by structural analysis. To accomplish this, we mutated contact residues involved in the PPI and assessed changes in binding affinities between mutants and wild-type proteins. We focused on the three main locations in the Cul2-VBC interface, namely: (1) the EloC pocket and adjacent area; (2) the helix α5-pVHL charge complementarity region; and (3) the hydrophobic contacts between helix α2 and EloC. In the EloC pocket area we mutated Leu3 to Ala to understand how important was the volume filling the EloC pocket for the interaction. We also mutated Leu3 to Gly, consistent with mutagenesis from previous work (Nguyen et al., 2015). Additionally we mutated Pro5 to Ala, as we considered that this residue could play a role in stabilizing the conformation of the N-terminal loop of Cul2, which seemed important to direct the interaction. Furthermore, Pro5 is involved in a three-way contact with Val181 (pVHL) and Met105 (EloC). In the other subunits, we mutated Val181 in pVHL to Gly and also mutated Met105 and Phe109 in EloC to Ala, to assess the consequences of disrupting the EloC hydrophobic pocket. In the helix α5 interface we mutated Gln111 and Lys114 to Leu and Glu, respectively, and Lys159 and Asp187 in pVHL to Glu and Lys, respectively, to understand the importance of these electrostatic interactions in the complex formation. Finally, in the third region of the interface we mutated residues Asn36, Phe39, Tyr43, and Val47 on Cul2 to Ala, as those residues are involved in hydrophobic contacts with pVHL and EloC surfaces.

Initially, we screened all the mutants generated in an AlphaLISA (Yasgar et al., 2016) assay to determine IC_{50} values from dose-response curves. To do this we designed a competition experiment in which the ability of a competitor (protein with mutation) to disrupt the interaction between the two native protein subunits of the complex is determined. VBC and Rbx1-Cul2 wild-type were also titrated for reference. The results of the AlphaLISA show that all the mutations are disruptive to the Cul2-VBC interaction (Figures 6 and S2), although the extent of the disruption varies greatly. We observed that the most destructive mutations, i.e., the ones that lead to a larger increase in the IC_{50} values, are mostly of residues located in the EloC pocket area. In particular, the mutation of Met105 and Phe109 increased the IC_{50} by 75- and 55-fold, respectively. Our results also show that K114E and K159E mutations in helix α5 interface do not interfere significantly with the IC_{50}, whereas Q111L and D187K

Table 2. Different Areas of Interface in Different Cullin-Rbx1 Complexes

Interaction Pair	Area of Interface (Å^2)	Rotation Angle (°)	Shift along the Axis (Å)	PDB
Rbx1-Cul5~ND8	1,707	124	10.2	3DQV
Rbx1-Cul2	2,122	–	–	5N4W
Rbx1-Cul5	2,307	118	−1.1	3DPL
Rbx1-Cul4	2,708	118	−1.9	4A0C
Rbx1-Cul1	3,396	114	−3.4	1U6G
Glomulin-Rbx1-Cul1	1,718	138	17.1	4F52

The areas of interface were calculated (PISA) as well as the rotation angle and shift along the rotation axis (Chimera), comparing the previously published structures with the RING domain in the new conformation.

have a more prominent effect. In the helix α2 interface, the mutations led to an increase in the IC_{50} values by about 3-fold. Based on these results, we picked some of the mutants for ITC experiments to determine K_D values. We selected the VBC mutants V181G, M105A, F109A, K159E, and D187K to quantify the importance of the hydrophobic environment in the EloC pocket and of the residues involved in the electrostatic interactions. In ITC (Table 3 and Figure S3) we observed the same trend as observed by AlphaLISA, with mutations M105A and F109A being the most disruptive and leading, in these conditions, to an increase of 20- and 37-fold in the K_D, respectively, which was accompanied by a significant loss of binding enthalpy. The mutations of V181G and D187K decreased the binding affinity by about 3-fold, whereas the mutation K159E resulted in a 5-fold decrease in the affinity.

Together, the biophysical data are consistent with a ranking of hotspots at the Cul2-VBC PPI, with the peripheral hydrophobic pocket composed of Met105 and Phe109 from EloC identified as the most critical among the mutations tested.

Swapping Residues and Selectivity for Cul2 or Cul5

Although explored in several publications (Kamura et al., 2004; Mahrour et al., 2008; Nguyen et al., 2015), the mechanism behind the selectivity between recruitment of Cul2 versus Cul5 by EloBC-containing CRL complexes remains partly elusive. One of the proposed hypotheses is that the electrostatic patch on pVHL surface is responsible for recruiting Cul2 (Nguyen et al., 2015); however, our data show that by inverting these two residues' charge, and therefore, inverting the electrostatics patch charge, VBC still binds to Cul2. To deepen our understanding on what drives this selectivity, we studied in parallel the $CRL5^{SOCS2}$ complex, one of the Cul5 ligases in which SOCS2 is the substrate receptor and EloBC is the adaptor subunit (SBC) (Bulatov et al., 2015; Bullock et al., 2006). Our choice was supported by the existence of a crystal structure of $Cul5_{NTD}$-SBC (Kim et al., 2013). By ITC we observed that, although using the same adaptor subunit, SBC does not bind Cul2. Intrigued by this observation, we investigated whether by mutating a combination of amino acid residues we could swap the selectivity of SBC with VBC for Cullin, or, in other words, if we could rescue the binding of SBC to Cul2 and, at the same time, decrease the affinity for Cul5 and vice versa. The structural analysis of Cul2-VBC versus Cul5-SBC (Figure 7A) reveals that Arg186 in SOCS2 would clash with Gln111 in Cul2, which would explain the lack of interaction observed, as this residue corresponds to a serine in pVHL (Ser183). It also highlights that Lys159 and Asp187 in pVHL correspond to Gln164 and Tyr190 in SOCS2, respectively. In SBC, Arg186 establishes hydrogen bonds with the backbone of Thr117 and with Gln113 in Cul5 but the other corresponding residues, while important for pVHL-Cul2 interaction, do not seem to be establishing any contact in this case (Figure 7A). Based on these observations, we generated triple mutants for VBC and SBC: in the first we mutated K159Q, S183R, D187Y and (V^{QRY}BC), and in the second we performed the reverse mutations: Q164K, R186S, and Y190D (S^{KSD}BC).

Initially we tested these variants in our AlphaLISA competition assay (Figure 7B), which suggested a switch in the recruitment. We observed not only a weakening of the interaction between V^{QRY}BC and Cul2 but also, pleasingly, that S^{KSD}BC rescued to some extent the binding to Cul2, compared with SBC. These data encouraged us to perform ITC experiments and evaluate the loss or gain in function, upon triple mutation (Figures 7C and 7D). For S^{KSD}BC, we observed a 25-fold loss in the binding

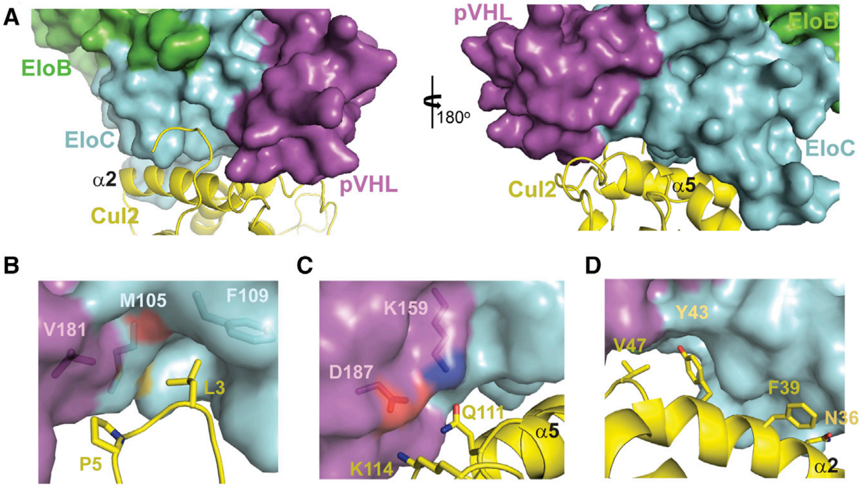

Figure 4. The Cul2-VBC Interface

(A) Contacts between $Cul2_{NTD}$ and VBC. As in similar CRL complexes, helix α2 of Cullin is the closest to the adaptor and substrate receptor subunits, establishing hydrophobic contacts.

(B) The N-terminal tail of Cul2 plays a substantial role in the PPI. Leu3 is accommodated in a hydrophobic pocket on the surface of EloC, and Pro5 is involved in a three-way interaction between Val181 from pVHL and Met105 from EloC.

(C) Residues Asp187 and Lys159 in pVHL establish an electrostatic network with residues Lys114 and Gln111 in helix α5 of Cul2.

(D) Residues Asn36, Phe39, Tyr43, and Val47 participate in hydrophobic contacts at the interface between helix α2 and EloC and pVHL.

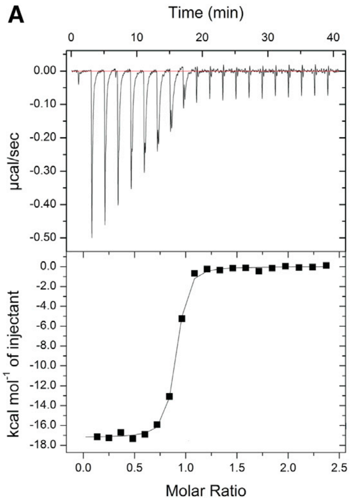

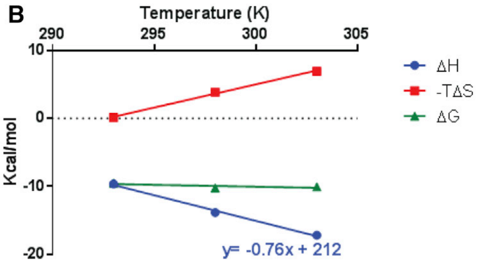

C

Complex	ΔCp (cal/mol/K)	Source
SOCS2-EloBC:Cul5$_{NTD}$	-450	Bulatov et al., 2015
Vif-EloBC:Cul5$_{NTD}$	-300	Salter et al., 2012
ASB9-EloBC:Cul5$_{NTD}$	-350	Thomas et al., 2013
VHL-EloBC:Cul2	-760	

Figure 5. Biophysical Characterization of the Interaction between VBC and Cul2

(A) ITC data whereby VBC (200 μM) was titrated into Rbx1-Cul2 (20 μM) at 303 K. Under these conditions, the binding affinity of the interaction (K_D) is 42 nM.

(B) Temperature dependency of the thermodynamic parameters ΔH, ΔG and $-T\Delta S$. The change in heating capacity, ΔC_p, was derived from the change in enthalpy with the temperature.

(C) Comparison of ΔC_p values for similar CRL systems.

affinity to Cul5 (K_D from 10 nM to 250 nM), whereas, on the other hand, we were able to partially rescue binding to Cul2 (K_D = 2.5 μM). Consistently, for V^{QRY}BC we observed total loss of binding to Cul2 upon triple mutation and partial rescue of binding to Cul5 (K_D = 2.9 μM). The rescued non-native interactions confirmed a switch in the ability to recruit Cullin, albeit with K_D values weaker than the ones measured for the native interactions (Figure 7E). Interestingly, the loss of binding affinity of S^{KSD}BC to Cul5 was accompanied by an increase in the enthalpy of the reaction, suggesting an interaction with a thermodynamic profile closer to that of Cul2-VBC (Figure S4).

Together, these findings shine light on the selectivity of VBC and SBC for Cul2 and Cul5, respectively, identifying amino acid positions that dictate this selectivity and mutations that contribute toward switching Cullin recruitment within these CRLs.

DISCUSSION

Ubiquitin ligases catalyze the transfer of ubiquitin to target proteins, and from this tagging event a variety of cellular responses can result. Progress has been achieved in recent years toward understanding the mechanisms underlying E3 ligase function, regulation, and ubiquitination activity (Buetow and Huang, 2016). CRLs in particular play important roles in cellular homeostasis by controlling the abundance of many regulatory proteins. This process is deregulated in many diseases including cancer, motivating the search for chemical probes targeting CRLs (Jia and Sun, 2011; Zhao and Sun, 2013). The emerging role of CRLs as targets for induced protein degradation by glue molecules (Petzold et al., 2016) and PROTACs (proteolysis targeting chimeras) (Deshaies, 2015) highlight their potential as therapeutic targets (Bulatov and Ciulli, 2015; Lamsoul et al., 2016). Despite the advances, detailed understanding at atomic level of the structure and function of whole CRL machines remains limited. Our new structure of the CRL2^{VHL} complex provides insights that contribute to the understanding of the ubiquitination mechanism. The structure highlights the flexibility of the central Cul2 scaffold, which has been observed in other Cullins (Goldenberg et al., 2004) and supports the existence of hinge bending within the protein. The hinge points identified underline this flexibility, which is deemed key for the ligase activity (Liu and Nussinov, 2011). Hinge points located in the linkers between the Cullin repeats allow the first and third helical bundles to come close or apart, alternating between an elongated and contracted shape. Another hinge point is located between the NTD and CTD. In our Cul2 structure the CTD is considerably tilted toward the NTD, in comparison with other CRL structures. Recent molecular dynamics studies have proposed a relationship between Cullin and Rbx1 dynamics and the ubiquitin chain length and topology built on the substrate (Onel et al., 2017). Structural

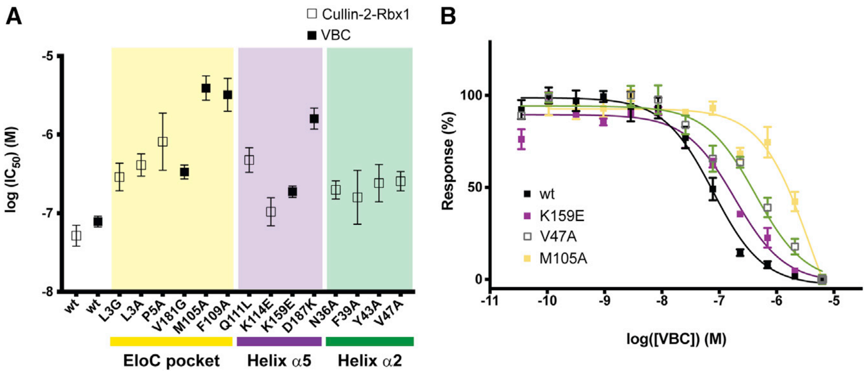

Figure 6. Residues in the EloC Hydrophobic Pocket Reveal Criticality for the Strong Binding Affinity of Cul2-VBC

(A) Plot of the log(IC_{50}) values resulting from the AlphaLISA competition experiment where mutant constructs of VBC or Cul2 were used to displace the native interaction between bead-bound wild-type protein. Mutations at the EloC pocket are highlighted in yellow, mutations at helix α5 interface are highlighted in purple, and mutations at helix α2 interface are highlighted in green. The fitting and calculation of IC_{50} values was performed with GraphPad Prism 7 software, and the error bars represent the error in the fitting function.

(B) Dose-response curves of the raw AlphaLISA data showing representative displacers from the three interaction areas in comparison with VBC wild-type (wt). The experiments were performed in quadruplicate and the results are an averaged value. The error bars represent the SD of each point.

Table 3. Isothermal Titration Calorimetry

VBC	K_D (nM)	Temperature (K)	ΔG (cal/mol)	ΔH (cal/mol)	$T\Delta S$ (cal/mol)	N
wt	10 ± 3	298	−10,958 ± 198	−12,700 ± 177	−1,742 ± 266	1
K159E	54 ± 9	298	−9,920 ± 94	−11,900 ± 174	−1,930 ± 198	0.9
D187K	27 ± 9	298	−10,339 ± 197	−11,300 ± 264	−961 ± 330	0.8
V181G	28 ± 7	298	−10,315 ± 154	−9,868 ± 174	447 ± 232	0.9
wt	9 ± 1	303	−11,017 ± 63	−14,800 ± 61	−3,783 ± 88	0.9
M105A	317 ± 39	303	−8,873 ± 74	−9,870 ± 141	−993 ± 159	0.9
F109A	177 ± 16	303	−9,221 ± 53	−9,260 ± 79	−41 ± 95	1

K_D values and thermodynamic parameters for the interactions between VBC variant proteins and Rbx1-Cul2 determined by ITC. VBC proteins (100 μM) were titrated into 10 μM Rbx1-Cul2. ITC titrations performed in the exact same conditions were compared. VBC M105A and F109A titrations were performed at 303 K to obtain better-quality data, as their interactions are of lower affinity, hence lower ΔH. The errors in the table reflect the quality of the fitting function.

studies of CRLs in complex with the COP9 signalosome have also highlighted the inherent Cullin flexibility (Cavadini et al., 2016; Mosadeghi et al., 2016). We present for the first time the interface between E2-recruiting RING, Rbx1, and Cul2, in which Rbx1 adopts a new orientation. Comparison with other Rbx1-Cullin structures suggests a pose in a trajectory from a closed to an open form; in other words, from inactive to active conformation of the complex.

CRLs must function as highly dynamic multi-subunit complexes. CRL flexibility is required to bring together substrate and ubiquitin that would otherwise be >50 Å apart, as found in some crystal structures (Zheng et al., 2002). Flexibility is also required to accommodate multiple attachment of ubiquitin at distinct positions in the chain during catalytic elongation cycles. The relative organization and dynamics of CRL subunits and their ability to work in concert are critical in achieving such flexibility. We consider that our new CRL2^{VHL} structure recapitulates many such dynamic features in addition to the intra- and the inter-subunit movements, observed in comparison with other CRL structures. For instance, the fact that we could not obtain crystals in the absence of the HIF-1α 19-mer peptide hints that the presence of the substrate may have been required to confer a conformational arrangement that facilitates crystallization. On the other hand, the lack of substantial electron density for the substrate-binding domain of pVHL suggests that this domain is highly flexible and might be present in more than one orientation in the crystal. Albeit not captured by the single-structure crystallographic model, this observation suggests functionally important dynamics

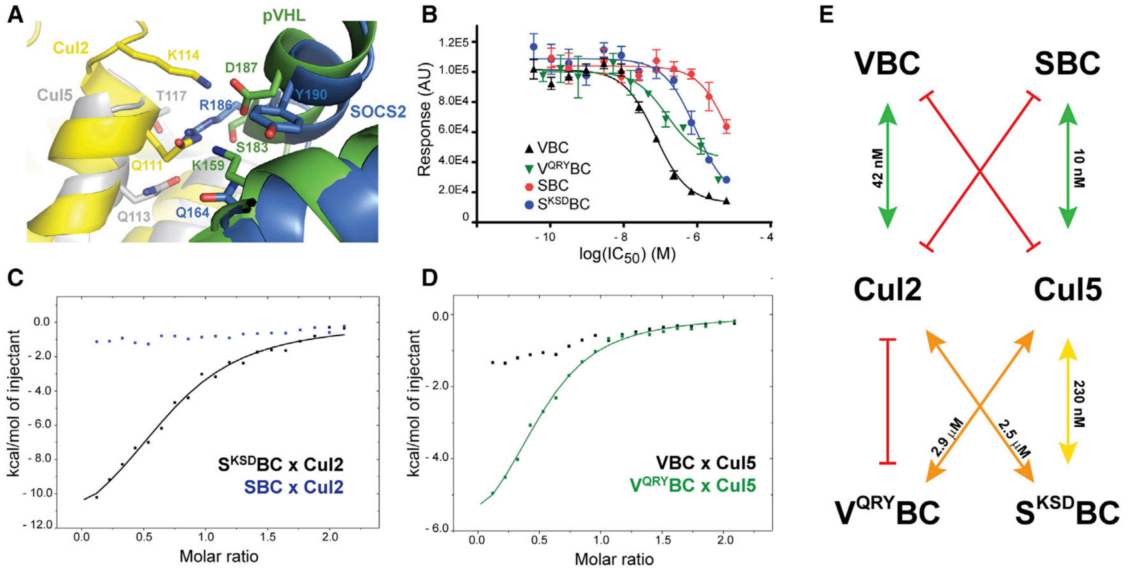

Figure 7. Swapping Residues and Selectivity for Cul2 or Cul5

(A) Structures of Cul5-SBC and Cul2-VBC aligned by the EloC subunit show residues involved in the electrostatic network created between substrate receptor and Cullin, in both cases.

(B) AlphaLISA data show loss of binding affinity of V^{QRY}BC toward Cul2 and rescue of binding of S^{KSD}BC toward Cul2. The experiments were performed in quadruplicate and the results are an averaged value. The error bars represent the SD of each point. The fitting was performed with GraphPad Prism 7 software.

(C and D) ITC data. Titrant solution (200 μM) was diluted into 20 μM titrate over 19 injections of 2 μL at 303 K. (C) Titration of SBC and S^{KSD}BC into Rbx1-Cul2. (D) Titration of Cul5$_{NTD}$ into V^{QRY}BC and VBC.

(E) Summary of the results obtained in the biophysical experiments.

of pVHL that may be driven by the presence of bound substrate. Work published by Nussinov and others (Liu and Nussinov, 2008; 2009) has elucidated pVHL's inter-domain flexibility by the presence of a linker region containing a conserved proline residue, which acts as a hinge and allows the rotation of the substrate-binding domain to position the substrate accurately for ubiquitination. The high average B factors observed after refinement for the overall structure also corroborate these dynamics. The resolution of our structure limits the information that can be extracted and, despite intensive efforts to improve the quality of the crystals, it was not possible to obtain a better-quality dataset.

We also report extensive biophysical data, which elucidate a key PPI interface of CRL complexes. The Cul2 interface with the adaptor and receptor subunits is conserved and characterized by a large buried surface area. The structural features are reflected in characteristic thermodynamic signatures, including enthalpy-driven interaction, tight binding affinity, and a large and negative change of heat capacity. We show that the interaction is contributed over extended regions, suggesting a spreading of hotspot residues across the interface. Emerging as largest contributors to the PPI affinity are hydrophobic residues in a distal pocket on EloC that recognizes the N-terminal tail of Cul2. This information could guide the design of molecules to target this specific region within $CRL2^{VHL}$.

Selectivity of Cullin recruitment in EloBC-containing complexes is an intriguing feature due to the similarities of the two PPIs in Cul2 and Cul5 CRLs. Kamura et al. (2004) were the first to demonstrate the importance of the entire Cul-box region (about 20 amino acids) of the substrate recognition subunit in determining the selectivity for Cul2 versus Cul5 recruitment. Here, by swapping only three topologically conserved residues at the receptor-scaffold interface in VBC and SBC, we were successful in modifying the ability of these proteins to bind Cul5 and Cul2, respectively. This result provides proof of concept for switching Cullin recruitment within CRLs by mutating individual amino acids, which, to our knowledge, is unprecedented. It could also lead to interesting applications for elucidating CRL biology and contribute to validating them as drug targets. It is likely that other residues could contribute to fine-tune Cullin selectivity, in addition to those identified by our work, which are not strictly conserved across all known Cul2 or Cul5 binders. For instance, it is known that the mutation of Trp53 in Cul5 results in a 30-fold loss of affinity to SBC (Kim et al., 2013). In Cul2, the corresponding residue to Trp53 is Ala48, and this could explain why the binding affinity of $V^{QRY}BC$ to Cul5 is not equal to the native Cul5-SBC interaction.

In conclusion, the new structural and biophysical data provided by our work shine new light on the structural assembly and dynamics functioning of CRLs and unveil mechanistic details and selectivity determinants for the Cul2-receptor PPI. It is anticipated that our findings will stimulate drug targeting of native, full-length CRL complexes, by aiding the development of novel chemical probes acting as either specific inhibitors or hijackers of CRL activity for targeted protein degradation.

STAR★METHODS

Detailed methods are provided in the online version of this paper and include the following:

- KEY RESOURCES TABLE
- CONTACT FOR REAGENT AND RESOURCE SHARING
- METHOD DETAILS
  - Expression and Purification of VBC, SBC and $Cul5_{NTD}$
  - Expression and Purification of Rbx1-Cul2
  - Site Directed Mutagenesis
  - Crystallization
  - Data Collection and Structure Solving
  - AlphaLISA
  - Isothermal Titration Calorimetry
- QUANTIFICATION AND STATISTICAL ANALYSIS
- DATA AND SOFTWARE AVAILABILITY
  - Data Resources

SUPPLEMENTAL INFORMATION

Supplemental Information includes four figures and six tables and can be found with this article online at http://dx.doi.org/10.1016/j.str.2017.04.009.

AUTHOR CONTRIBUTIONS

A.C., T.A.F.C., and M.S.G. designed experiments. T.A.F.C. performed experiments. T.A.F.C. and M.S.G. solved the crystal structure. A.C. and T.A.F.C. wrote the manuscript.

ACKNOWLEDGMENTS

This work was supported by the European Research Council ERC-2012-StG-311460 DrugE3CRLs (Starting Grant to A.C.), the Fundação para a Ciência e a Tecnologia SFRH/BD/92417/2013 (PhD Studentship to T.A.F.C.), and the Wellcome Trust (strategic awards 100476/Z/12/Z and 094090/Z/10/Z to the Division of BCDD). We thank Dr. A. Knebel and C. Johnson for sharing their protocols of expression and purification of Rbx1-Cul2, Dr. M. Peggie for the Cul2-Rbx1 plasmid, and S. Shepherd and the PPT for access to tissue culture facilities and advice. We are also thankful to Dr. P. Fyfe for support with the in-house X-ray facility and to Diamond Light Source for beamtime (BAG proposal MX10071-48) and beamline support at beamline I04-1.

Received: February 28, 2017
Revised: April 7, 2017
Accepted: April 28, 2017
Published: June 6, 2017

REFERENCES

Adams, P.D., Afonine, P.V., Bunkóczi, G., Chen, V.B., Davis, I.W., Echols, N., Headd, J.J., Hung, L.-W., Kapral, G.J., Grosse-Kunstleve, R.W., et al. (2010). PHENIX: a comprehensive Python-based system for macromolecular structure solution. Acta Crystallogr. D Biol. Crystallogr. *66*, 213–221.

Angers, S., Li, T., Yi, X., MacCoss, M.J., Moon, R.T., and Zheng, N. (2006). Molecular architecture and assembly of the DDB1-CUL4A ubiquitin ligase machinery. Nature *443*, 590–593.

Babon, J.J., Sabo, J.K., Zhang, J.-G., Nicola, N.A., and Norton, R.S. (2009). The SOCS box encodes a hierarchy of affinities for Cullin5: implications for ubiquitin ligase formation and cytokine signalling suppression. J. Mol. Biol. *387*, 162–174.

Buckley, D.L., Raina, K., Darricarrere, N., Hines, J., Gustafson, J.L., Smith, I.E., Miah, A.H., Harling, J.D., and Crews, C.M. (2015). HaloPROTACS: use of small molecule PROTACs to induce degradation of HaloTag fusion proteins. ACS Chem. Biol. *10*, 1831–1837.

Buetow, L., and Huang, D.T. (2016). Structural insights into the catalysis and regulation of E3 ubiquitin ligases. Nat. Rev. Mol. Cell Biol. *17*, 626–642.

Bulatov, E., and Ciulli, A. (2015). Targeting Cullin-RING E3 ubiquitin ligases for drug discovery: structure, assembly and small-molecule modulation. Biochem. J. *467*, 365–386.

Bulatov, E., Martin, E.M., Chatterjee, S., Knebel, A., Shimamura, S., Konijnenberg, A., Johnson, C., Zinn, N., Grandi, P., Sobott, F., et al. (2015). Biophysical studies on interactions and assembly of full-size E3 ubiquitin ligase: suppressor of cytokine signaling 2 (SOCS2)-elongin BC-cullin 5-ring box protein 2 (RBX2). J. Biol. Chem. *290*, 4178–4191.

Bullock, A.N., Debreczeni, J.E., Edwards, A.M., Sundström, M., and Knapp, S. (2006). Crystal structure of the SOCS2-elongin C-elongin B complex defines a prototypical SOCS box ubiquitin ligase. Proc. Natl. Acad. Sci. USA *103*, 7637–7642.

Cai, W., and Yang, H. (2016). The structure and regulation of Cullin 2 based E3 ubiquitin ligases and their biological functions. Cell Div. *11*, 7.

Calabrese, M.F., Scott, D.C., Duda, D.M., Grace, C.R.R., Kurinov, I., Kriwacki, R.W., and Schulman, B.A. (2011). A RING E3-substrate complex poised for ubiquitin-like protein transfer: structural insights into cullin-RING ligases. Nat. Struct. Mol. Biol. *18*, 947–949.

Canning, P., Cooper, C.D.O., Krojer, T., Murray, J.W., Pike, A.C.W., Chaikuad, A., Keates, T., Thangaratnarajah, C., Hojzan, V., Ayinampudi, V., et al. (2013). Structural basis for Cul3 protein assembly with the BTB-Kelch family of E3 ubiquitin ligases. J. Biol. Chem. *288*, 7803–7814.

Cavadini, S., Fischer, E.S., Bunker, R.D., Potenza, A., Lingaraju, G.M., Goldie, K.N., Mohamed, W.I., Faty, M., Petzold, G., Beckwith, R.E.J., et al. (2016). Cullin-RING ubiquitin E3 ligase regulation by the COP9 signalosome. Nature *531*, 598–603.

Chen, V.B., Arendall, W.B., Headd, J.J., Keedy, D.A., Immormino, R.M., Kapral, G.J., Murray, L.W., Richardson, J.S., and Richardson, D.C. (2010). MolProbity: all-atom structure validation for macromolecular crystallography. Acta Crystallogr. D Biol. Crystallogr. *66*, 12–21.

Deshaies, R.J. (2015). Protein degradation: prime time for PROTACs. Nat. Chem. Biol. *11*, 634–635.

Duda, D.M., Borg, L.A., Scott, D.C., Hunt, H.W., Hammel, M., and Schulman, B.A. (2008). Structural insights into NEDD8 activation of cullin-RING ligases: conformational control of conjugation. Cell *134*, 995–1006.

Duda, D.M., Scott, D.C., Calabrese, M.F., Zimmerman, E.S., Zheng, N., and Schulman, B.A. (2011). Structural regulation of cullin-RING ubiquitin ligase complexes. Curr. Opin. Struct. Biol. *21*, 257–264.

Duda, D.M., Olszewski, J.L., Tron, A.E., Hammel, M., Lambert, L.J., Waddell, M.B., Mittag, T., DeCaprio, J.A., and Schulman, B.A. (2012). Structure of a glomulin-RBX1-CUL1 complex: inhibition of a RING E3 ligase through masking of its E2-binding surface. Mol. Cell *47*, 371–382.

Emsley, P., and Cowtan, K. (2004). Coot: model-building tools for molecular graphics. Acta Crystallogr. D Biol. Crystallogr. *60*, 2126–2132.

Evans, P.R., and Murshudov, G.N. (2013). How good are my data and what is the resolution? Acta Crystallogr. D Biol. Crystallogr. *69*, 1204–1214.

Fischer, E.S., Scrima, A., Böhm, K., Matsumoto, S., Lingaraju, G.M., Faty, M., Yasuda, T., Cavadini, S., Wakasugi, M., Hanaoka, F., et al. (2011). The molecular basis of CRL4DDB2/CSA ubiquitin ligase architecture, targeting, and activation. Cell *147*, 1024–1039.

Fraczkiewicz, R., and Braun, W. (1998). Exact and efficient analytical calculation of the accessible surface areas and their gradients for macromolecules. J. Comput. Chem. *19*, 319–333.

Frost, J., Galdeano, C., Soares, P., Gadd, M.S., Grzes, K.M., Ellis, L., Epemolu, O., Shimamura, S., Bantscheff, M., Grandi, P., et al. (2016). Potent and selective chemical probe of hypoxic signalling downstream of HIF-α hydroxylation via VHL inhibition. Nat. Commun. *7*, 13312.

Fulcher, L.J., Macartney, T., Bozatzi, P., Hornberger, A., Rojas-Fernandez, A., and Sapkota, G.P. (2016). An affinity-directed protein missile system for targeted proteolysis. Open Biol. *6*, 160255.

Galdeano, C., Gadd, M.S., Soares, P., Scaffidi, S., Van Molle, I., Birced, I., Hewitt, S., Dias, D.M., and Ciulli, A. (2014). Structure-guided design and optimization of small molecules targeting the protein-protein interaction between the von Hippel-Lindau (VHL) E3 ubiquitin ligase and the hypoxia inducible factor (HIF) alpha subunit with in vitro nanomolar affinities. J. Med. Chem. *57*, 8657–8663.

Goldenberg, S.J., Cascio, T.C., Shumway, S.D., Garbutt, K.C., Liu, J., Xiong, Y., and Zheng, N. (2004). Structure of the Cand1-Cul1-Roc1 complex reveals regulatory mechanisms for the assembly of the multisubunit cullin-dependent ubiquitin ligases. Cell *119*, 517–528.

Hershko, A., and Ciechanover, A. (1998). The ubiquitin system. Annu. Rev. Biochem. *67*, 425–479.

Hon, W.C., Wilson, M.I., Harlos, K., Claridge, T.D.W., Schofield, C.J., Pugh, C.W., Maxwell, P.H., Ratcliffe, P.J., Stuart, D.I., and Jones, E.Y. (2002). Structural basis for the recognition of hydroxyproline in alpha IF-1 alpha by pVHL. Nature *417*, 975–978.

Jia, L., and Sun, Y. (2011). SCF E3 ubiquitin ligases as anticancer targets. Curr. Cancer Drug Targets *11*, 347–356.

Kamura, T., Koepp, D.M., Conrad, M.N., Skowyra, D., Moreland, R.J., Iliopoulos, O., Lane, W.S., Kaelin, W.G., Elledge, S.J., Conaway, R.C., et al. (1999). Rbx1, a component of the VHL tumor suppressor complex and SCF ubiquitin ligase. Science *284*, 657–661.

Kamura, T., Maenaka, K., Kotoshiba, S., Matsumoto, M., Kohda, D., Conaway, R.C., Conaway, J.W., and Nakayama, K.I. (2004). VHL-box and SOCS-box domains determine binding specificity for Cul2-Rbx1 and Cul5-Rbx2 modules of ubiquitin ligases. Genes Dev. *18*, 3055–3065.

Keegan, R.M., and Winn, M.D. (2007). Automated search-model discovery and preparation for structure solution by molecular replacement. Acta Crystallogr. D Biol. Crystallogr. *63*, 447–457.

Kelsall, I.R., Duda, D.M., Olszewski, J.L., Hofmann, K., Knebel, A., Langevin, F., Wood, N., Wightman, M., Schulman, B.A., and Alpi, A.F. (2013). TRIAD1 and HHARI bind to and are activated by distinct neddylated Cullin-RING ligase complexes. EMBO J. *32*, 2848–2860.

Kim, Y.K., Kwak, M.-J., Ku, B., Suh, H.-Y., Joo, K., Lee, J., Jung, J.U., and Oh, B.-H. (2013). Structural basis of intersubunit recognition in elongin BC-cullin 5-SOCS box ubiquitin-protein ligase complexes. Acta Crystallogr. D Biol. Crystallogr. *69*, 1587–1597.

Lai, A.C., and Crews, C.M. (2017). Induced protein degradation: an emerging drug discovery paradigm. Nat. Rev. Drug Discov. *16*, 101–114.

Lamsoul, I., Uttenweiler-Joseph, S., Moog-Lutz, C., and Lutz, P.G. (2016). Cullin 5-RING E3 ubiquitin ligases, new therapeutic targets? Biochimie *122*, 339–347.

Lee, D.W., Peggie, M., Deak, M., Toth, R., Gage, Z.O., Wood, N., Schilde, C., Kurz, T., and Knebel, A. (2012). The Dac-tag, an affinity tag based on penicillin-binding protein 5. Anal. Biochem. *428*, 64–72.

Liu, J., and Nussinov, R. (2008). Allosteric effects in the marginally stable von Hippel-Lindau tumor suppressor protein and allostery-based rescue mutant design. Proc. Natl. Acad. Sci. USA *105*, 901–906.

Liu, J., and Nussinov, R. (2009). The mechanism of ubiquitination in the cullin-RING E3 ligase machinery: conformational control of substrate orientation. PLoS Comput. Biol. *5*, e1000527.

Liu, J., and Nussinov, R. (2011). Flexible cullins in cullin-RING E3 ligases allosterically regulate ubiquitination. J. Biol. Chem. *286*, 40934–40942.

Lu, J., Qian, Y., Altieri, M., Dong, H., Wang, J., Raina, K., Hines, J., Winkler, J.D., Crew, A.P., Coleman, K., et al. (2015). Hijacking the E3 ubiquitin ligase cereblon to efficiently target BRD4. Chem. Biol. *22*, 755–763.

Lucas, X., and Ciulli, A. (2017). Recognition of substrate degrons by E3 ubiquitin ligases and modulation by small-molecule mimicry strategies. Curr. Opin. Struct. Biol. *44*, 101–110.

Lydeard, J.R., Schulman, B.A., and Harper, J.W. (2013). Building and remodelling Cullin-RING E3 ubiquitin ligases. EMBO Rep. *14*, 1050–1061.

Mahrour, N., Redwine, W.B., Florens, L., Swanson, S.K., Martin-Brown, S., Bradford, W.D., Staehling-Hampton, K., Washburn, M.P., Conaway, R.C., and Conaway, J.W. (2008). Characterization of Cullin-box sequences that direct recruitment of Cul2-Rbx1 and Cul5-Rbx2 modules to Elongin BC-based ubiquitin ligases. J. Biol. Chem. *283*, 8005–8013.

Matthews, B.W. (1968). Solvent content of protein crystals. J. Mol. Biol. *33*, 491–497.

Maxwell, P.H., Wiesener, M.S., Chang, G.W., Clifford, S.C., Vaux, E.C., Cockman, M.E., Wykoff, C.C., Pugh, C.W., Maher, E.R., and Ratcliffe, P.J.

(1999). The tumour suppressor protein VHL targets hypoxia-inducible factors for oxygen-dependent proteolysis. Nature *399*, 271–275.

McCoy, A.J., Grosse-Kunstleve, R.W., Adams, P.D., Winn, M.D., Storoni, L.C., and Read, R.J. (2007). Phaser crystallographic software. J. Appl. Crystallogr. *40*, 658–674.

Min, J.-H., Yang, H., Ivan, M., Gertler, F., Kaelin, W.G., and Pavletich, N.P. (2002). Structure of an HIF-1alpha-pVHL complex: hydroxyproline recognition in signaling. Science *296*, 1886–1889.

Mosadeghi, R., Reichermeier, K.M., Winkler, M., Schreiber, A., Reitsma, J.M., Zhang, Y., Stengel, F., Cao, J., Kim, M., Sweredoski, M.J., et al. (2016). Structural and kinetic analysis of the COP9-Signalosome activation and the cullin-RING ubiquitin ligase deneddylation cycle. Elife *5*, e12102.

Muchnik, E., and Kaplan, J. (2011). HIF prolyl hydroxylase inhibitors for anemia. Expert Opin. Investig. Drugs *20*, 645–656.

Muniz, J.R.C., Guo, K., Kershaw, N.J., Ayinampudi, V., von Delft, F., Babon, J.J., and Bullock, A.N. (2013). Molecular architecture of the ankyrin SOCS box family of Cul5-dependent E3 ubiquitin ligases. J. Mol. Biol. *425*, 3166–3177.

Nguyen, H.C., Yang, H., Fribourgh, J.L., Wolfe, L.S., and Xiong, Y. (2015). Insights into cullin-RING E3 ubiquitin ligase recruitment: structure of the VHL-EloBC-Cul2 complex. Structure *23*, 441–449.

Nicholls, R.A., Long, F., and Murshudov, G.N. (2012). Low-resolution refinement tools in REFMAC5. Acta Crystallogr. D Biol. Crystallogr. *68*, 404–417.

Onel, M., Sumbul, F., Liu, J., Nussinov, R., and Haliloglu, T. (2017). Cullin neddylation may allosterically tune polyubiquitin chain length and topology. Biochem. J. *474*, 781–795.

Pause, A., Lee, S., Worrell, R.A., Chen, D.Y., Burgess, W.H., Linehan, W.M., and Klausner, R.D. (1997). The von Hippel-Lindau tumor-suppressor gene product forms a stable complex with human CUL-2, a member of the Cdc53 family of proteins. Proc. Natl. Acad. Sci. USA *94*, 2156–2161.

Petroski, M.D., and Deshaies, R.J. (2005). Function and regulation of cullin-RING ubiquitin ligases. Nat. Rev. Mol. Cell Biol. *6*, 9–20.

Pettersen, E.F., Goddard, T.D., Huang, C.C., Couch, G.S., Greenblatt, D.M., Meng, E.C., and Ferrin, T.E. (2004). UCSF Chimera–a visualization system for exploratory research and analysis. J. Comput. Chem. *25*, 1605–1612.

Petzold, G., Fischer, E.S., and Thomä, N.H. (2016). Structural basis of lenalidomide-induced CK1α degradation by the CRL4(CRBN) ubiquitin ligase. Nature *532*, 127–130.

Prabhu, N.V., and Sharp, K.A. (2005). Heat capacity in proteins. Annu. Rev. Phys. Chem. *56*, 521–548.

Salter, J.D., Lippa, G.M., Belashov, I.A., and Wedekind, J.E. (2012). Core-binding factor β increases the affinity between human Cullin 5 and HIV-1 Vif within an E3 ligase complex. Biochemistry *51*, 8702–8704.

Scott, D.C., Sviderskiy, V.O., Monda, J.K., Lydeard, J.R., Cho, S.E., Harper, J.W., and Schulman, B.A. (2014). Structure of a RING E3 trapped in action reveals ligation mechanism for the ubiquitin-like protein NEDD8. Cell *157*, 1671–1684.

Semenza, G.L. (2007). Life with oxygen. Science *318*, 62–64.

Spratt, D.E., Wu, K., Kovacev, J., Pan, Z.-Q., and Shaw, G.S. (2012). Selective recruitment of an E2∼ubiquitin complex by an E3 ubiquitin ligase. J. Biol. Chem. *287*, 17374–17385.

Stebbins, C.E., Kaelin, W.G., and Pavletich, N.P. (1999). Structure of the VHL-ElonginC-ElonginB complex: implications for VHL tumor suppressor function. Science *284*, 455–461.

Thomas, J.C., Matak-Vinkovic, D., Van Molle, I., and Ciulli, A. (2013). Multimeric complexes among ankyrin-repeat and SOCS-box protein 9 (ASB9), ElonginBC, and Cullin 5: insights into the structure and assembly of ECS-type Cullin-RING E3 ubiquitin ligases. Biochemistry *52*, 5236–5246.

Vagin, A.A., Steiner, R.A., Lebedev, A.A., Potterton, L., McNicholas, S., Long, F., and Murshudov, G.N. (2004). REFMAC5 dictionary: organization of prior chemical knowledge and guidelines for its use. Acta Crystallogr. D Biol. Crystallogr. *60*, 2184–2195.

Van Molle, I., Thomann, A., Buckley, D.L., So, E.C., Lang, S., Crews, C.M., and Ciulli, A. (2012). Dissecting fragment-based lead discovery at the von Hippel-Lindau protein:hypoxia inducible factor 1α protein-protein interface. Chem. Biol. *19*, 1300–1312.

Winn, M.D., Ballard, C.C., Cowtan, K.D., Dodson, E.J., Emsley, P., Evans, P.R., Keegan, R.M., Krissinel, E.B., Leslie, A.G.W., McCoy, A., et al. (2011). Overview of the CCP4 suite and current developments. Acta Crystallogr. D Biol. Crystallogr. *67*, 235–242.

Winter, G.E., Buckley, D.L., Paulk, J., Roberts, J.M., Souza, A., Dhe-Paganon, S., and Bradner, J.E. (2015). Phthalimide conjugation as a strategy for in vivo target protein degradation. Science *348*, 1376–1381.

Yasgar, A., Jadhav, A., Simeonov, A., and Coussens, N.P. (2016). AlphaScreen-based assays: ultra-high-throughput screening for small-molecule inhibitors of challenging enzymes and protein-protein interactions. Methods Mol. Biol. *1439*, 77–98.

Zengerle, M., Chan, K.-H., and Ciulli, A. (2015). Selective small molecule induced degradation of the BET bromodomain protein BRD4. ACS Chem. Biol. *10*, 1770–1777.

Zhao, Y., and Sun, Y. (2013). Cullin-RING Ligases as attractive anti-cancer targets. Curr. Pharm. Des. *19*, 3215–3225.

Zheng, N., Schulman, B.A., Song, L., Miller, J.J., Jeffrey, P.D., Wang, P., Chu, C., Koepp, D.M., Elledge, S.J., Pagano, M., et al. (2002). Structure of the Cul1-Rbx1-Skp1-F boxSkp2 SCF ubiquitin ligase complex. Nature *416*, 703–709.

STAR★METHODS

KEY RESOURCES TABLE

REAGENT or RESOURCE	SOURCE	IDENTIFIER
Bacterial and Virus Strains		
Escherichia coli BL21(DE3)	Alessio Ciulli Lab	N/A
Escherichia coli DH10Bac	Alessio Ciulli Lab	N/A
Chemicals, Peptides, and Recombinant Proteins		
Celfectin II	ThermoFisher Scientific	10362100
KOD Hotstart polymerase	EMD Millipore	71086
DpnI enzyme	New England Biolabs	R0176S
Critical Commercial Assays		
Biotinylation kit EZ-link NHS-biotin	ThermoFisher Scientific	20217
Deposited Data		
Coordinated and structure factors have been deposited in the Protein Data Bank	This study	PDB ID: 5N4W
Experimental Models: Organisms/Strains		
Insect cells: Spodoptera Frugiperda 21	MRC-PPU, Dundee	N/A
Oligonucleotides		
Primers used for site-directed mutagenesis can be consulted in Table S6	Sigma Aldrich	N/A
Recombinant DNA		
pVHL plasmid (to express His-tagged pVHL$_{54-213}$)	Alessio Ciulli Lab	pIVM02
SOCS2 plasmid (to express His-tagged SOCS2$_{32-198}$)	SGC	SOCS2A-c016
EloB$_{1-104}$/EloC$_{17-112}$ plasmid (to co-express with pVHL or SOCS2)	Alessio Ciulli Lab	pIVM26
Cullin-5$_{1-386}$ plasmid (to express His-tagged Cul5$_{NTD}$)	SGC	CUL5a-c001
Rbx1–Cullin-2 plasmid (pFastBacDUAL, for expression of full-length Rbx1 and DAC-tagged Cullin-2)	MRC-PPU, Dundee	DU23263
Software and Algorithms		
Aimless	(Evans and Murshudov, 2013)	http://www.ccp4.ac.uk/html/aimless.html
Refmac5	(Vagin et al., 2004)	http://www.ccp4.ac.uk/html/refmac5/description.html
Phenix	(Adams et al., 2010)	https://www.phenix-online.org/
Coot	(Emsley and Cowtan, 2004)	https://www2.mrc-lmb.cam.ac.uk/personal/pemsley/coot/
Molprobity	(Chen et al., 2010)	http://molprobity.biochem.duke.edu/
GetArea	(Fraczkiewicz and Braun, 1998)	http://curie.utmb.edu/getarea.html
Chimera	(Pettersen et al., 2004)	https://www.cgl.ucsf.edu/chimera/download.html
MicroCal Origin 7.0	Malvern Instruments	N/A
GraphPad Prism Software	GraphPad Software, Inc.	https://www.graphpad.com/scientific-software/prism/
Other		
Anti-6xHis AlphaLISA acceptor beads	Perkin Elmer	AL128M
Streptavidin AlphaLISA donor beads	Perkin Elmer	6760002

CONTACT FOR REAGENT AND RESOURCE SHARING

Further information and requests for resources and reagents should be directed to and will be fulfilled by the Lead Contact, Alessio Ciulli (a.ciulli@dundee.ac.uk)

METHOD DETAILS

Expression and Purification of VBC, SBC and $Cul5_{NTD}$

VBC and SBC ternary complexes and variant proteins were purified as described previously (Bulatov et al., 2015; Van Molle et al., 2012). BL21(DE3) *E. coli* cells were co-transformed with the plasmid for expression of pVHL/SOCS2 and the plasmid for expression of Elongin B and Elongin C. A single colony of transformant was used to inoculate LB media for bacterial culture. Protein expression was induced with 0.3 mM IPTG at 24°C for 18 hours. Co-expression of these proteins resulted in the formation of the respective trimeric complex (VBC/SBC) that was then purified by affinity chromatography, followed by ion-exchange chromatography and finally by size-exclusion chromatography. Following this protocol the yield of protein was about 15-20 mg per litre of culture.

$Cul5_{NTD}$ (residues 1–386) was also expressed in BL21(DE3) *E. coli* cells (Thomas et al., 2013). After transformation and inoculation of LB media for bacterial growth, protein expression was induced with 0.5 mM IPTG at 18°C for 18 hours. His-tagged $Cul5_{NTD}$ was purified by affinity chromatography and by size-exclusion chromatography with a yield of ~40 mg of protein per litre of culture.

Expression and Purification of Rbx1-Cul2

Rbx1-Cul2 containing an N-terminal Dac-tag (Lee et al., 2012) in Cul2 was expressed in *Sf21* insect cells. The recombinant bacmid and the resulting recombinant baculovirus were generated using protocols adapted from the *Bac-to-Bac®* system. *Sf21* cells at a density of $1.5x10^6$ cells/ml were infected with the P1 virus in a 1:100 ratio and incubated at 27°C, 135 rpm, in the dark for 72 hours. The cells were harvested by centrifugation, the pellet was re-suspended in lysis buffer containing 50 mM HEPES pH 8.0, 250 mM NaCl, 2 mM TCEP and 0.2% Triton-X and the cells were lysed by French press. The lysate was clarified by centrifugation and the supernatant was mixed with ampicillin-modified sepharose resin. After 1 hour incubation at room temperature, the resin was washed three times with 20 mM HEPES pH 8.0, 100 mM NaCl, 5% glycerol, 2 mM TCEP. At the last wash step the resin was suspended in the same buffer and incubated with TEV enzyme for 2.5 hours at room temperature. The cleaved Rbx1-Cul2 was recovered through filtration and the filtrate was loaded on a Superdex 200 gel filtration column (GE Healthcare) after concentration for further purification.

The protein's identities were confirmed by electrospray mass spectrometry analysis.

Site Directed Mutagenesis

pVHL, SOCS2, Elongin C and Cul2 mutants were prepared by PCR-based method using the respective expression vectors encoding for the wild type proteins as template. The amplification of the expression vectors was performed using the KOD hot-start DNA polymerase (EMD Millipore), following the manufacturer guidelines and specific pairs of primers (Table S6) were used for the introduction of the desired mutation. PCR products were treated with DpnI enzyme (New England Biolabs) and transformed in DH5α *E.coli* cells. The mutations were confirmed by DNA sequencing.

Crystallization

VBC was incubated with an HIF-1α 19-mer peptide (residues 559-557) and the resulting complex (VBCH) was purified on a Superdex 75 gel filtration column (GE Healthcare). VBCH and Rbx1-Cul2 were mixed in equimolar ratio and incubated for 30 min at room temperature. The $CRL2^{VHL}$ complex was buffer-exchanged to 50 mM Tris-HCl pH 8.0, 150 mM NaCl, 5 mM DTT in a Superdex 200 Increase gel filtration column (GE Healthcare). The pentameric complex was concentrated to 4.2 mg/ml. Equal volumes of $CRL2^{VHL:HIF}$ complex and liquor solution were mixed in the hanging-drop vapour diffusion method at 20°C. The liquor solution contained 0.1 M Tris pH 7.6, 0.15 M ammonium sulfate, 15% polyethyleneglycol 4000 and 3% 1,4-dioxane or 4% acetonitrile as additive. After equilibration, the drop was streaked with seeds of disrupted $CRL2^{VHL:HIF}$ crystals. Crystals would generally appear within 48 hours. Crystals were cryoprotected with 20% ethyleneglycol or 20% glycerol and screened using an in-house Rigaku M007HF X-ray generator and Saturn 944HG+ CCD detector.

Data Collection and Structure Solving

X-ray data were collected at 100 K and a wavelength of 0.9282 Å at Diamond Light Source beamline I04-1. Indexing and integration of reflections was performed using DIALS, and scaling and merging with AIMLESS in CCP4i (Evans and Murshudov, 2013; Winn et al., 2011). The Cul2-VBC interface was modeled taking advantage of the higher resolution structure available [PDB 4WQO, (Nguyen et al., 2015)]. The isomorphous dataset was refined using REFMAC5 (Vagin et al., 2004) and COOT (Emsley and Cowtan, 2004). MOLPROBITY (Chen et al., 2010) server was used to validate the geometry and steric clashes in the structures.

AlphaLISA

For the AlphaLISA experiments Anti-6xHis acceptor beads and Streptavidin donor beads (PerkinElmer) were used. Competition assay was performed in a 384-well plate by mixing $V_{6xHis}BC$ (500 nM) and biotinylated Rbx1-Cul2 (EZ-link NHS-biotin ThermoFisher Scientific) (150 nM) and the competitor in a concentration range from 6.25 μM to 35 nM (final concentration). The mixture was incubated for 1 hour at room temperature. Next, the anti-6xHis beads were added to the mixture in the dark and the mixture was incubated for another hour. Finally, the streptavidin beads were added, followed by another hour of incubation. The final volume of each well was 20 μl. The plate was then read in a PHERAstar FS (BMG LABTECH). Each of the competitors was titrated in quadruplicate.

Isothermal Titration Calorimetry

Isothermal titration calorimetry (ITC) experiments were carried in an ITC_{200} microcalorimeter (Malvern). VBC (wild type and variant proteins), SBC (wild type and variant proteins) were titrated into Rbx1-Cul2. For the experiments with $Cul5_{NTD}$ the latter was titrated against VBC (wild type and variant proteins) or SBC (wild type and variant proteins). Titrations consisted of 19 injections of 2 μl each (120 seconds spacing and 600 rpm stirring speed). All protein solutions were dialysed into 100 mM Bis tris propane pH 8.0, 50 mM NaCl, 2 mM TCEP prior to the titrations. Control experiments were performed subtracted to the relevant experiment to account for heat of dilution. Data analyses for the ITC experiments were performed using the MicroCal Origin 7.0 software package. Binding enthalpy, dissociation constants, and stoichiometry were determined by fitting the data using a one-set-of-sites binding model. The experiments were performed twice for consistency.

QUANTIFICATION AND STATISTICAL ANALYSIS

Statistical details of experiments can be found in the figure and table legends. ITC experiments were performed in duplicate. The data was fitted using the one-set-of-sites model in Origin 7.0 software. AlphaLISA experiments were performed in quadruplicate and the fitting was performed using the average ± standard deviation. The fitting of the data was performed using GraphPad Prism 7.

DATA AND SOFTWARE AVAILABILITY

Data Resources

Coordinates and structure factors have been deposited in the Protein Data Bank (PDB) with the accession code 5N4W.

Structure, Volume *25*

Supplemental Information

Crystal Structure of the Cul2-Rbx1-EloBC-VHL Ubiquitin Ligase Complex

Teresa A.F. Cardote, Morgan S. Gadd, and Alessio Ciulli

Supplemental Information

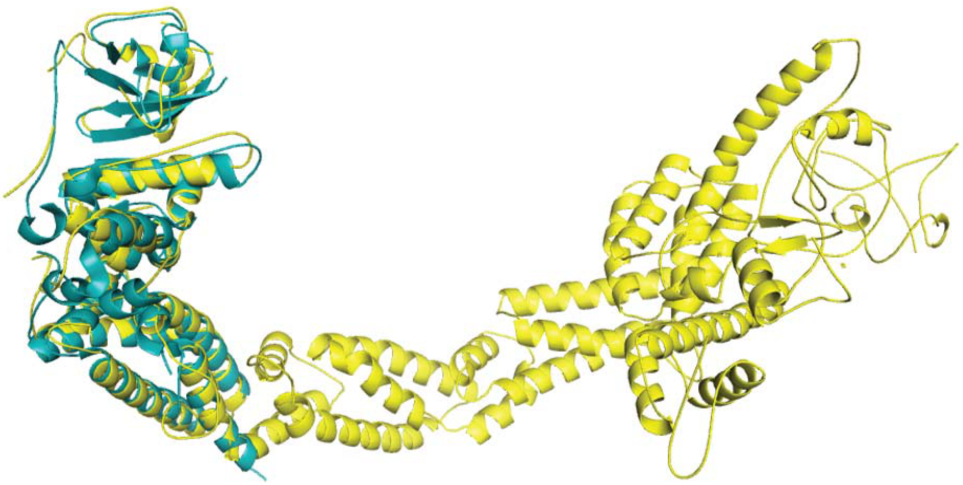

Figure S1. Related to Figure 1. Superposition of Cul2$_{1-163}$–VBC and CRL2^{VHL}. – The structure of the CRL2^{VHL} complex (PDB 5N4W) is superposed with the structure of VBC bound to Cul2$_{NTD}$ (PDB 4WQO). The structures align with a rmsd of 0.866 Å over the Cα atoms.

Table S1. Related to Figure 1. – Comparison of the areas of interface between Cul2 and VBC in the two structures available.

	**Buried surface area involving Cul2 interfaces (Å^2)**		
**PDB**	**pVHL-Cul2**	**EloC-Cul2**	**Total**
4WQO	322	944	1270
5N4W	328	1022	1350

Table S2. Related to Figure 5. – Buried surface area in the cullin interface with the receptor domains in three CRL complexes. PDBs 5N4W, 4JGH and 4N9F for $CRL2^{VHL}$, $CRL5^{SOCS2}$ and $CRL5^{Vif}$, respectively. The areas were calculated with GetArea (Fraczkiewicz & Braun 1998).

	Area (Å^2)		
	Polar	Apolar	Total
VBC	5556	7915	13471
Cul2	17339	26443	43782
Cul2-VBC	21997	32635	54632
ΔSASA	-899	-1723	-2621
SBC	7270	10909	18179
$Cul5_{NTD}$	7710	11709	19418
$Cul5_{NTD}$-SBC	14361	21291	35652
ΔSASA	-619	-1326	-1945
VifCβFBC	10068	14102	24170
$Cul5_{NTD}$	6540	9737	16278
$Cul5_{NTD}$-VifCβFBC	16085	22757	38842
ΔSASA	-523	-1082	-1605

Table S3. Related to Figure 5. – Comparison between experimental and theoretical Δ*Cp* values (Prabhu & Sharp 2005) for the Cul2–VBC interaction.

	Source	Δ*Cp* (cal/mol/K)
Experimental		-760
Theoretical	Spolar et al.	-425
	Murphy & Friere	-540
	Myers et al.	-400
	Makhatadze & Privalov	-692
	Robertson & Murphy	-384

Figure S2. Related to Figure 6. – AlphaLISA raw data of the titration of VBC and Cul2 mutants as displacers. The experiments were performed in quadruplicate and the results are an averaged value. The error bars represent the standard deviation of each point. The fitting was performed with GraphPad Prism 7 software.

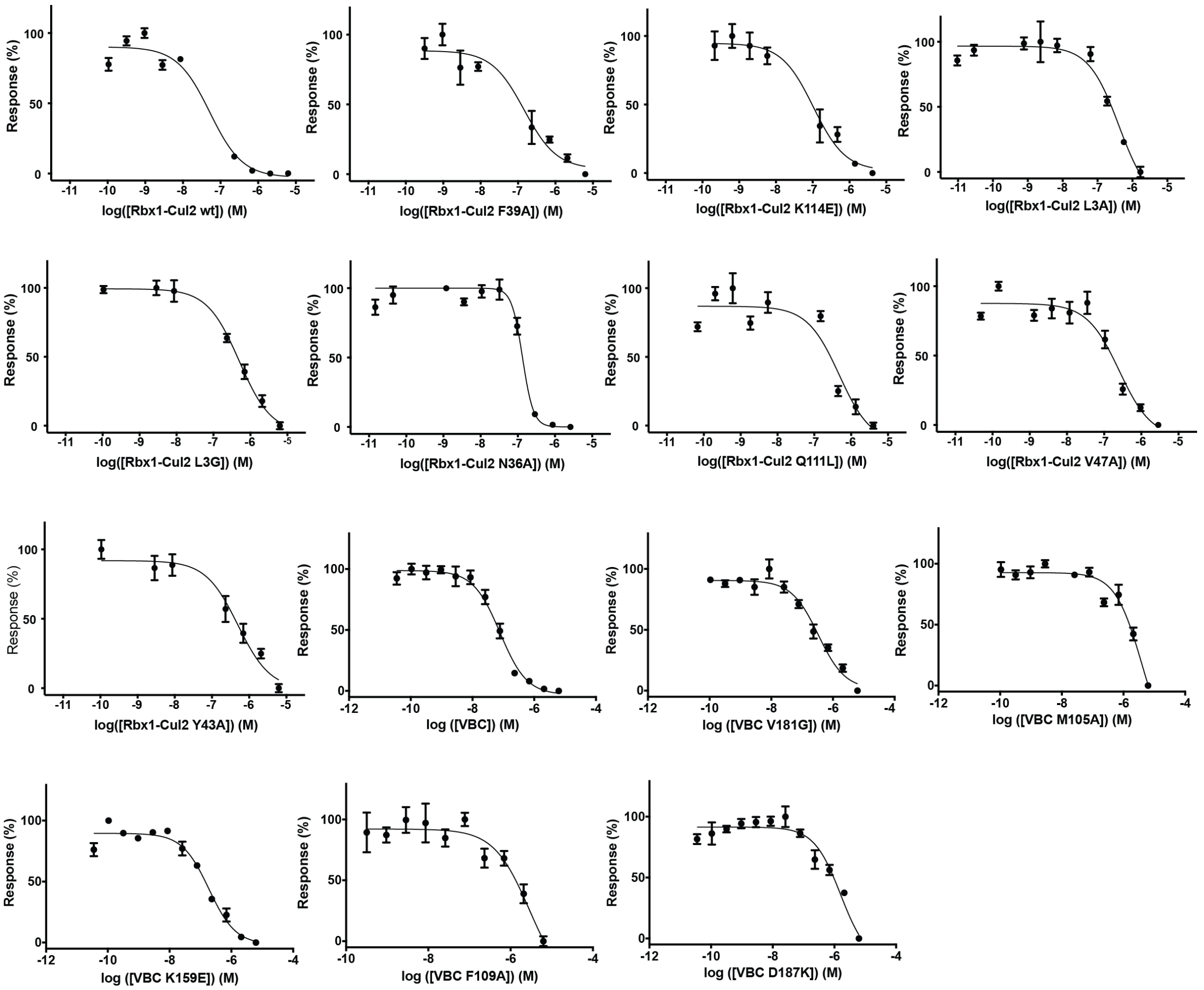

Table S4. Related to Figure 6 and Figure S2. – Alphalisa data of the titration of VBC and Cul2 mutants as displacers.

Displacer	IC_{50} (M)	95% CI	R^2	pIC_{50} (M)
VBC wt	7.80E-08	5.65E-08 to 1.08E-07	0.9550	-7.11 ± 0.07
VBC K159E	1.87E-07	1.34E-07 to 2.63E-07	0.9504	-6.73 ± 0.07
VBC D187K	1.59E-06	8.52E-07 to 2.97E-06	0.8773	-5.80 ± 0.13
VBC V181G	3.34E-07	2.21E-07 to 5.05E-07	0.9339	-6.48 ± 0.09
VBC M105A	3.90E-06	1.89E-06 to 8.02E-06	0.8978	-5.41 ± 0.15
VBC F109A	2.89E-06	1.40E-06 to 5.94E-06	0.8796	-5.54 ± 0.15
Cullin-2 wt	5.16E-08	2.77E-08 to 9.63E-08	0.9629	-7.29 ± 0.13
Cullin-2 L3A	4.08E-07	2.11E-07 to 7.91E-07	0.8816	-6.39 ± 0.14
Cullin-2 L3G	2.88E-07	1.26E-07 to 6.57E-07	0.8353	-6.54 ± 0.18
Cullin-2 P5A	8.11E-07	1.46E-07 to 4.50E-06	0.8302	-6.09 ± 0.36
Cullin-2 N36A	1.97E-07	1.16E-07 to 3.36E-07	0.9000	-6.71 ± 0.11
Cullin-2 F39A	1.59E-07	5.14E-08 to 4.91E-07	0.7952	-6.80 ± 0.24
Cullin-2 Y43A	2.40E-07	7.94E-08 to 7.27E-07	0.7529	-6.62 ± 0.24
Cullin-2 V47A	2.54E-07	1.43E-07 to 4.51E-07	0.8869	-6.59 ± 0.12
Cullin-2 Q111L	4.74E-07	2.27E-07 to 9.87E-07	0.8509	-6.32 ± 0.16
Cullin-2 K114E	1.04E-07	4.51E-08 to 2.41E-07	0.8798	-6.98 ± 0.18

Figure S3. Related to Table 3. – Isothermal Titration Calorimetry raw data of the titrations of VBC mutants versus Rbx1–Cul2.

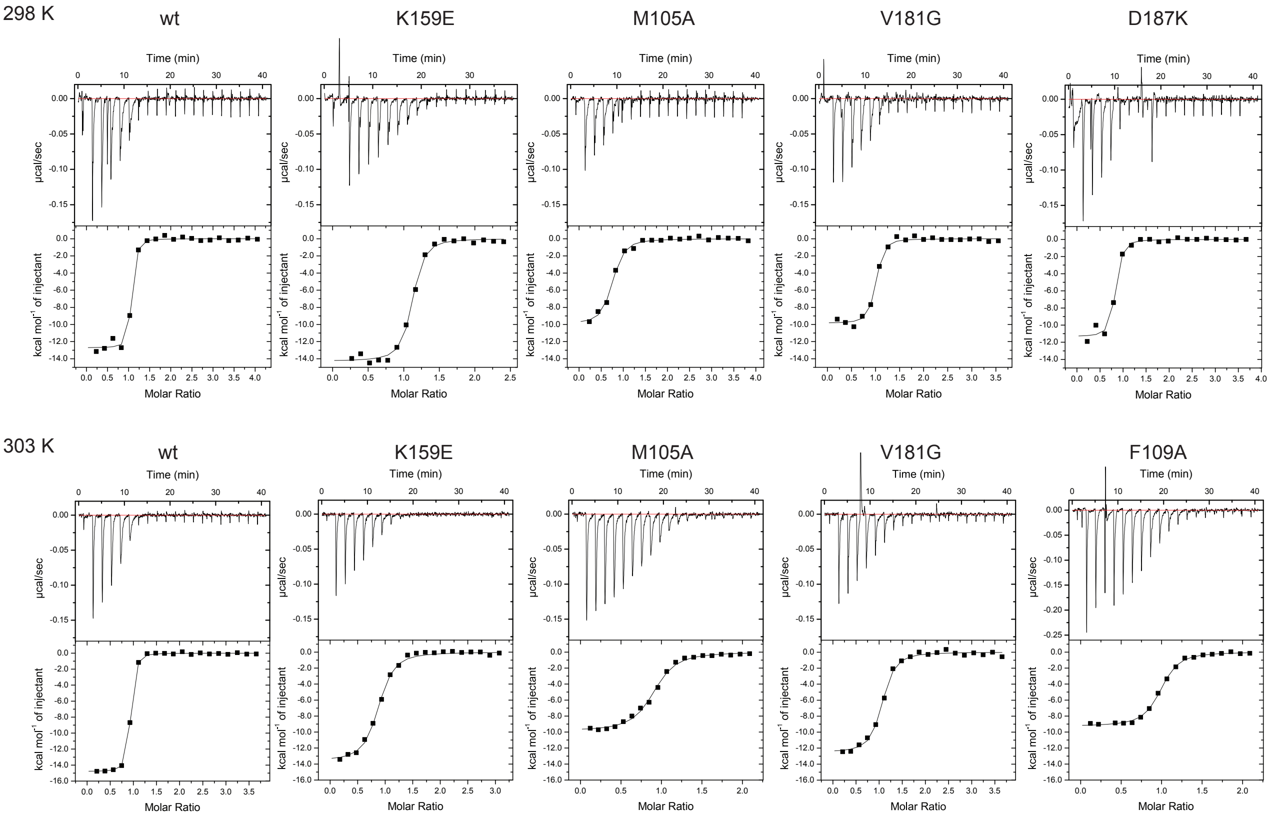

Figure S4. Related to Figure 7. Isothermal titration calorimetry data. – 200 μM of titrant diluted into 20 μM of titrate over 19 injections of 2 μl at 303 K. **a)** Titration of $Cul5_{NTD}$ into SBC and $S^{KSD}BC$. **b)** Titration of $V^{QRY}BC$ and VBC into Rbx1-Cul2.

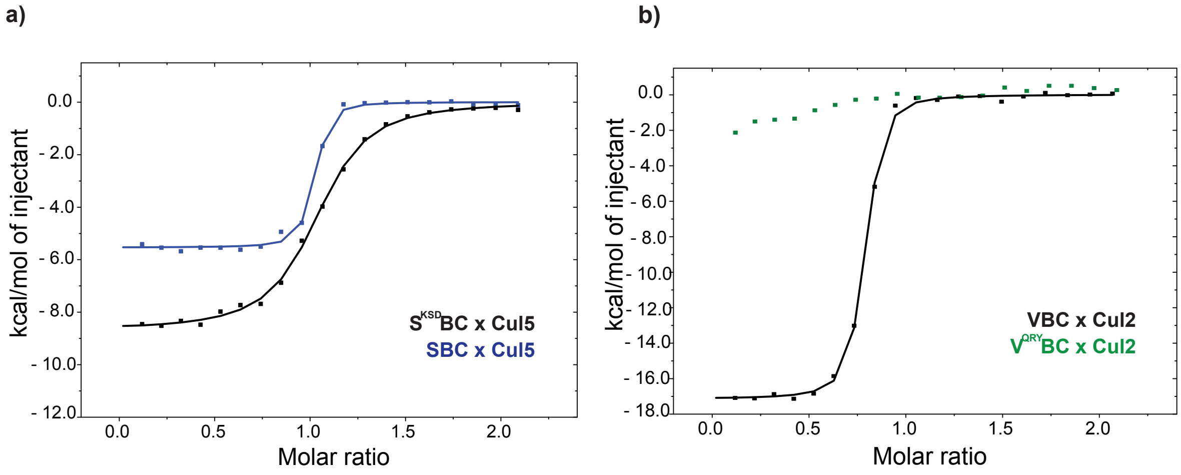

Table S5. Related to Figure 7 and Figure S4. – Isothermal Titration Calorimetry processed data of the experiments of the swapped domains. 150 μM of titrant diluted into 15 μM of titrate over 19 injections of 2 μl at 303 K. The errors refer to the fitting function.

	K_D (M)			ΔG (cal/mol)			ΔH (cal/mol)			TΔS (cal/mol)		
VBC x Cul2	4.2×10^{-8}	±	5.0×10^{-9}	-10068	±	70	-17210	±	113	-7142	±	133
V^{QRY}BC x Cul2	ND			ND			ND			ND		
SBC x Cul2	ND			ND			ND			ND		
S^{KSD}BC x Cul2	2.5×10^{-6}	±	2.7×10^{-7}	-7652	±	64	-12980	±	524	-5328	±	528
VBC X Cul5	ND			ND			ND			ND		
V^{QRY}BC x Cul5	2.9×10^{-6}	±	3.0×10^{-7}	-7554	±	59	-7400	±	386	154	±	39
SBC x Cul5	9.8×10^{-9}	±	2.1×10^{-9}	-10936	±	124	-5161	±	33	5775	±	128
S^{KSD}BC x Cul5	2.3×10^{-7}	±	3.2×10^{-8}	-9058	±	81	-8354	±	109	704	±	136

Table S6. Related to STAR Methods. – Primers used for the mutations in Cullin-2, pVHL, Elongin C and SOCS2.

Protein	Mutation	Forward primer 5' -> 3'	Reverse primer 5'->3'
Cullin-2	L3A	CAGGGCGGATCCATGTCTGCGAAACCAAGAGTAGTAG	CTACTACTCTTGGTTTCGCAGACATGGATCCGCCCTG
	L3G	GTTCCGCGTGGATCCATGTCTGGGAAACCAAGAGTAGTAG	CTACTACTCTTGGTTTCCCAGACATGGATCCACGCGGAAC
	P5A	CCGCGTGGATCCATGTCTTTGAAAGCAAGAGTAGTAGATTTTGATGAAACATGG	CCATGTTTCATCAAAATCTACTACTCTTGCTTTCTCAGACATGGATCCACGCGG
	N36A	GGAATACGTCGAAAGAGCAACATGGGCTGACCGTTTCTCAGATATCTATGCTTTATG	CATAAAGCATAGATATCTGAGAAACGGTCAGCCCATGTTGCTCTTTCGACGTATTCC
	F39A	CGTCGAAAGAGCAACATGGAATGACCGTGCCTCAGATATCTATGCTTTATGTGTGGCC	GGCCACACATAAAGCATAGATATCTGAGGCACGGTCATTCCATGTTGCTCTTTCGACG
	Y43A	GGAATGACCGTTTCTCAGATATCGCTGCTTTATGTGTGGCCTATCCTGAACCCC	GGGGTTCAGGATAGGCCACACATAAAGCAGCGATATCTGAGAAACGGTCATTCC
	V47A	CAGATATCTATGCTTTATGTGCGGCCTATCCTGAACCCCTTGGAG	CTCCAAGGGGTTCAGGATAGGCCGCACATAAAGCATAGATATCTG
	Q111L	GGTATCTCAACACCCTGTTTATTAAAAAGAATAAATTAACAGAAGCGGACC	GGTCCGCTTCTGTTAATTTATTCTTTTTAATAAACAGGGTGTTGAGATACC
	K114E	GTATCTCAACACCCAGTTTATTGAAAAGAATAAATTAACAGAAG	CTTCTGTTAATTTATTCTTTTCAATAAACTGGGTGTTGAGATAC
pVHL	K159E	CACTGCCAGTGTATACTCTGGAAGAGCGATGCCTCCAGGTTGTCC	GGACAACCTGGAGGCATCGCTCTTCCAGAGTATACACTGGCAGTG
	K159Q	CCAATTGAGATCCCTAACAATCTAAGGCGTTCGCAGCCATCAGCAGTTCC	CCAATTGAGATCCCTAACAATCTAAGGCGTTCGCAGCCATCAGCAGTTCC
	V181G	GAATTACAGGAGACTGGACATCGGCAGGTCGCTCTACGAAGATC	GATCTTCGTAGAGCGACCTGCCGATGTCCAGTCTCCTGTAATTC
	S183R+D187Y	CCAATTGAGATCCCTAACAATCTAAGGCGTTCGCAGCCATCAGCAGTTCC	CGTCAGGCGGCTCTACGAATATCTGGAAGACCACCCAAATGTGC
	D187K	CATCGTCAGGTCGCTCTACGAAAAACTGGAAGACCACCCAAATG	CATTTGGGTGGTCTTCCAGTTTTTCGTAGAGCGACCTGACGATG
EloC	M105A	GAAATTGCACTGGAACTGCTGGCGGCTGCGAACTTCTTAGATTGTTAG	CTAACAATCTAAGAAGTTCGCAGCCGCCAGCAGTTCCAGTGCAATTTC
	F109A	GGAACTGCTGATGGCTGCGAACGCCTTAGATTGTTAGGGATCTCAATTGG	CCAATTGAGATCCCTAACAATCTAAGGCGTTCGCAGCCATCAGCAGTTCC
SOCS2	Q164K	CCGCTCTACACGTCAGCACCATCTCTGAAGCATCTCTGTAGGCTCACCATTAAC	GTTAAGTGTGAGCCTACAGAGATGCTTCAGAGATGGTGCTGACGTGTAGAGCGG
	R186S+Y190D	CATCTGGGGACTGCCTTTACCAACAAGCCTAAAAGATGACTTGGAAG	CTTCCAAGTAATCTTTTAGGCTTGTTGGTAAAGGCAGTCCCCAGATG
